# Direct-Acting Antivirals and Risk of Hepatitis C Extrahepatic Manifestations

**DOI:** 10.1001/jamanetworkopen.2025.14631

**Published:** 2025-06-11

**Authors:** Dahn Jeong, Stanley Wong, Mohammad Ehsanul Karim, Amee R. Manges, Jean Damascene Makuza, Héctor Alexander Velásquez García, Prince Asumadu Adu, Mawuena Binka, Amanda Yu, Sofia R. Bartlett, Mel Krajden, Naveed Zafar Janjua

**Affiliations:** 1 School of Population and Public Health, University of British Columbia, Vancouver, British Columbia, Canada; 2 British Columbia Centre for Disease Control, Vancouver, British Columbia, Canada; 3 Centre for Advancing Health Outcomes, St Paul’s Hospital, Vancouver, British Columbia, Canada; 4 Department of Pathology and Laboratory Medicine, University of British Columbia, Vancouver, British Columbia, Canada; 5 University of British Columbia Centre for Disease Control, University of British Columbia, Vancouver, British Columbia, Canada

## Abstract

**Importance:**

Hepatitis C virus (HCV) infection is associated with various extrahepatic manifestations (EHMs) that can significantly impact patients’ quality of life and overall health outcomes.

**Objective:**

To assess the association between successful direct-acting antiviral (DAA) treatment and the risk of EHMs in individuals with chronic HCV infection.

**Design, Setting, and Participants:**

This population-based retrospective cohort study used data from 1990 to 2021, with a median follow-up of 2.5 years for outcome assessment. People treated with DAAs were matched to those who were never treated by year of HCV diagnosis and inverse probability of treatment weights (IPTW) were estimated to adjust for differences in baseline characteristics between treated and untreated people. The study took place in British Columbia, Canada, using the BC Hepatitis Testers Cohort, which includes more than 1 300 000 people tested for HCV between 1990 to 2015. Data were analyzed from February 2024 to March 2025.

**Exposure:**

Successful HCV treatment with DAAs, defined as achieving sustained virologic response (SVR).

**Main Outcomes and Measures:**

Incidence of 5 groups of EHMs: chronic kidney disease and end-stage kidney disease (CKD and ESKD), type 2 diabetes (T2D), stroke, major adverse cardiac events (MACE), and neurocognitive disorders (NCD).

**Results:**

Of 22 576 individuals included in this study (mean [SD] age at HCV diagnosis, 42.0 [12.0] years; 14 950 male [66.2%]), 11 953 received DAA treatment and achieved SVR, 386 received treatment but did not achieve SVR, and 10 237 never received treatment. The incidence rates (per 1000 person-years) in untreated vs treated with SVR groups were: 21.0 (95% CI, 19.0-23.1) vs 14.7 (95% CI, 13.4-16.1) for CKD and ESKD, 8.9 (95% CI, 7.7-10.2) vs 6.3 (95% CI, 5.5-7.2) for stroke, 26.7 (95% CI, 24.5- 29.1) vs 19.3 (95% CI, 17.8-20.9) for MACE, 19.2 (95% CI, 17.3-21.2) vs 10.3 (95% CI, 9.2-11.5) for NCD, and 6.4 (95% CI, 5.4-7.7) vs 9.2 (95% CI, 8.1-10.4) for T2D. In multivariable IPTW-weighted regression models, successful DAA treatment was associated with lower risk of CKD and ESKD (adjusted cause-specific hazard ratio [acsHR], 0.54; 95% CI, 0.47-0.63), stroke (acsHR, 0.66; 95% CI, 0.54-0.81), MACE (acsHR, 0.58; 95% CI, 0.52-0.66), and NCD (acsHR, 0.52; 95% CI, 0.45-0.61) compared with no treatment. However, no significant association was found for T2D (acsHR, 1.04; 95% CI, 0.84-1.29).

**Conclusions and Relevance:**

In this cohort study of 22 576 individuals with chronic HCV infection, successful HCV treatment with DAAs was associated with lower risk of several EHMs. These findings highlight the importance of early HCV screening and treatment to prevent EHMs and improve overall health outcomes.

## Key Points

**Question:**

Is there an association between successful treatment of chronic hepatitis C virus (HCV) infection with direct-acting antivirals (DAAs) and the risk of extrahepatic manifestations?

**Findings:**

In this population-based cohort study of 22 576 individuals with chronic HCV infection in British Columbia, Canada, successful DAA treatment was associated with lower risk of chronic kidney disease, stroke, major adverse cardiac events, and neurocognitive disorders compared with no treatment. However, it was not associated with reduced risk of type 2 diabetes.

**Meaning:**

These findings suggest that successful HCV treatment with DAAs was associated with lower risk of several important extrahepatic manifestations, underscoring the importance of early HCV screening and treatment to improve overall health outcomes.

## Teaser

This cohort study examines successful direct-acting antiviral treatment and the risk of extrahepatic manifestations among individuals with chronic hepatitis C virus infections from 1990 to 2021.

## Introduction

Despite significant advancements in diagnostic and treatment strategies, chronic HCV infection continues to be a major public health challenge globally. Alongside its well-known progression to liver damage, cirrhosis, and liver cancer,^1^ HCV infection has been associated with nonhepatic complications, known as extrahepatic manifestations (EHMs). These include immune disorders, neurological manifestations, kidney, cardiovascular, cerebrovascular diseases, and type 2 diabetes (T2D).^2^ The pathophysiological mechanisms between HCV and EHMs are complex, with evidence suggesting that viral replication in extrahepatic cells, heightened immune reaction causing systemic effects and chronic inflammatory state may contribute to EHMs.^3,4,5,6^ EHMs exacerbate the HCV disease sequelae and have a profound impact on individuals’ quality of life. To improve overall health of people who have or had chronic HCV infection, a deeper understanding of the association between HCV treatment and the risks of EHMs is needed.

Historically, HCV infection was managed with interferon (IFN)-based therapies, which were associated with severe adverse effects and virologic cure rates between 40% to 60%.^7^ Despite their lower cure rates, successful treatment of HCV with IFN-based therapies has been associated with decreased risk of EHMs.^8,9,10,11^ The introduction of direct-acting antivirals (DAAs) in recent years has transformed HCV treatment, presenting a significant opportunity to further optimize HCV care and mitigate the burden of extrahepatic complications. In Canada and British Columbia, DAAs were first approved in 2014 but with restrictive eligibility criteria (fibrosis stage, F2 or higher).^12^ Since 2018, DAAs have been fully subsidized without restrictions in British Columbia under the publicly funded provincial plan for prescription drugs.^13^

Studies following the advent of DAAs suggested that HCV cure achieved through DAA treatment may be associated with reduction of EHMs.^14^ However, many of these studies were constrained by a small sample size,^15^ focused on specific patient cohort,^16^ or lacked an untreated comparison group.^17^ Additionally, while existing studies have examined the association between DAAs and cardiovascular and cerebrovascular outcomes, there has been limited investigation into chronic kidney diseases and neurocognitive disorders. To advance our understanding of the protective effects of DAAs against extrahepatic manifestations, it is critical to assess diverse EHMs, particularly within large, population-based cohorts.

This study aimed to evaluate the association between successful HCV treatment with DAAs and the risk of EHMs, leveraging data from a large, population-based, linked administrative dataset from British Columbia, Canada. We examined the incidence of EHMs among individuals who received DAAs and achieved sustained virologic response (SVR), as well as those who had not, compared with individuals who never received HCV treatment.

## Methods

### Data Source

The British Columbia Hepatitis Testers Cohort (BC-HTC) includes more than 1 300 000 people tested for HCV from 1990 to 2015. This cohort is linked with various administrative health datasets and PharmaNet drug dispensation data, including all DAA prescriptions, through a unique personal health number (eTable 1 in Supplement 1).^18^ For this analysis, linked administrative data were updated to March 31, 2021. The study was approved by the University of British Columbia’s Behavioral Research Ethics Board. This cohort study followed the Strengthening the Reporting of Observational Studies in Epidemiology (STROBE) reporting guideline. Consent was not required because this was a secondary analysis of deidentified data.

### Study Population, Design, and Exposure

Study population included individuals diagnosed with chronic HCV by March 31, 2020. DAA treatment was defined as filling 1 or more DAA prescription. Sustained virologic response (SVR) was determined by negative HCV RNA test 10 or more weeks posttreatment, with most people being assessed SVR at 12 or more weeks posttreatment. Individuals were classified as treated and SVR, treated and no SVR, or untreated. We excluded individuals with spontaneous clearance, those who received only IFN-based therapies, and those without adequate follow-up for SVR assessment.

To adjust for the immortal time bias between people who were treated and those who were never treated, we used prescription-time distribution matching,^19^ randomly matching each treated person to an untreated person, by the year of their first HCV RNA diagnosis, within a 12-month time frame, at a 1:1 ratio without replacement. The SVR assessment date for people who received treatment was considered as the index date, and we assigned the same index date to the matched untreated person, to equalize the period of outcome observation between those who received treatment and those who were never treated.

**Figure 1.**
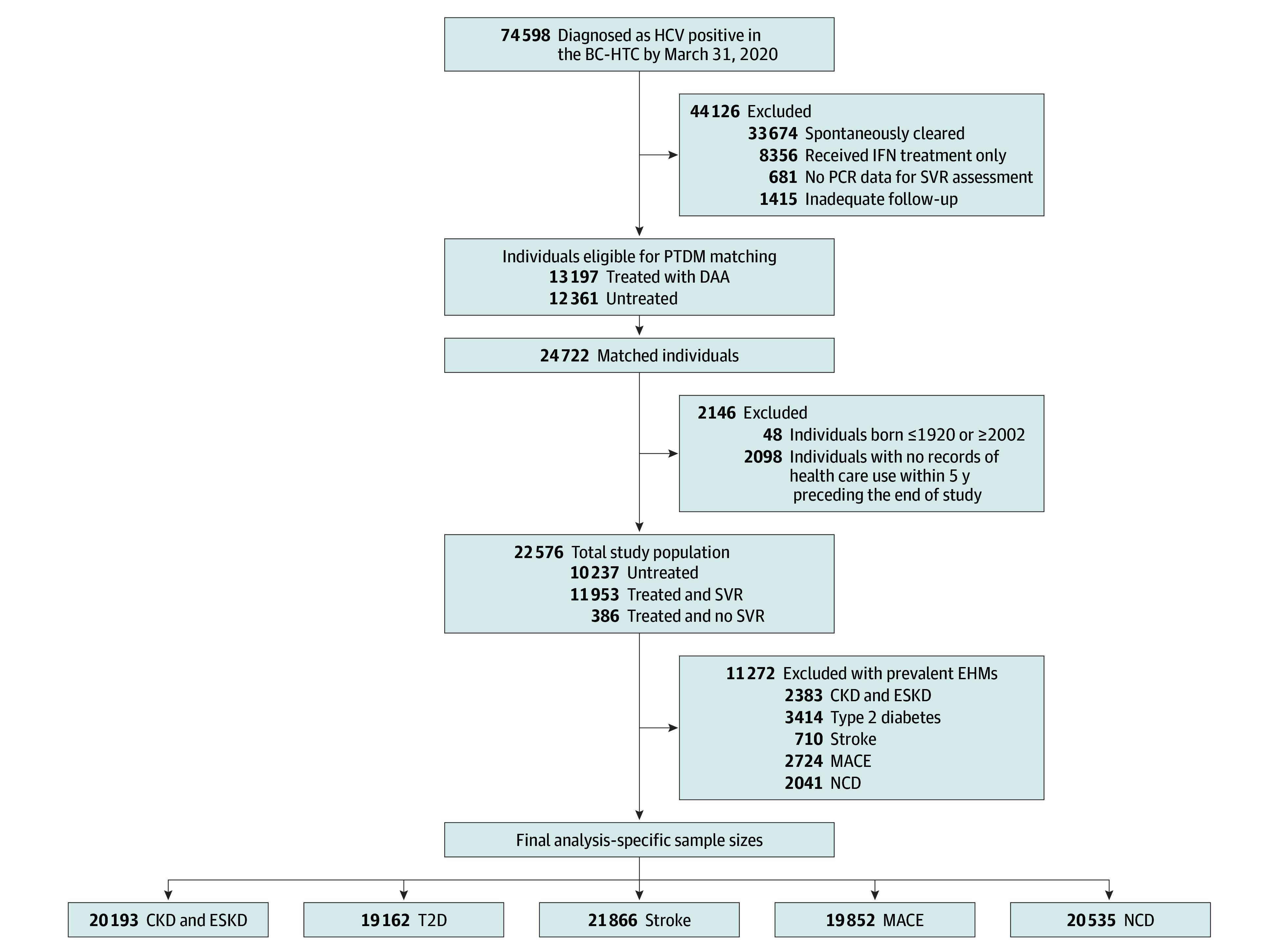
Study Inclusion Flowchart for Assessing the Association Between DAA Treatment and Incident EHMs BC-HTC, British Columbia Hepatitis Testers Cohort; CKD, chronic kidney disease; DAA, direct-acting antivirals; EHM, extrahepatic manifestations; ESKD, end-stage kidney disease; HCV, hepatitis C virus; IFN, interferon; MACE, major adverse cardiac events; NCD, neurocognitive disorders; SVR, sustained virologic response; PCR, polymerase chain reaction; PTDM, prescription-time distribution matching; T2D, type 2 diabetes.

Furthermore, individuals who had no records of health care use during 5 years preceding the end of study were excluded as the outcome assessment in this study was based on records in health administrative datasets. Figure 1 illustrates the details on exclusions and steps to generating the final analysis-specific study samples.

### Outcome Assessment

We categorized EHMs into 5 groups^6,11^: (1) chronic kidney diseases (CKD) and end-stage kidney diseases (ESKD); (2) T2D; (3) hospitalized stroke, including hemorrhagic, ischemic stroke and transient ischemic attack (TIA) (hereafter grouped as stroke); (4) major adverse cardiac events (MACE), including acute myocardial infarction, angina, heart failure, peripheral vascular disease, percutaneous transluminal coronary angioplasty, coronary artery bypass graft and stroke; and (5) neurocognitive disorders (NCD), including dementia, delirium and Alzheimer disease. These were defined using the Canadian *International Classification of Diseases, Ninth *and *Tenth Revisions, Canada* diagnostic and procedural codes (eTable 2 in Supplement 1). EHMs occurring prior to the index date were considered prevalent and excluded from analysis. EHMs were assessed until March 31, 2021.

### Variable Selection

Based on previous literature, we considered the following clinical and sociodemographic variables as risk factors for EHMs^2,20,21^: sex; age; ethnicity; material and social deprivation quintiles; HCV genotype; HBV infection; HIV infection; history of hypertension, statin use, obesity, nonalcoholic fatty liver disease, cirrhosis, major mental illness, alcohol use disorder, drug dependence disorder, injection drug use, opioid agonist therapy; and Elixhauser Comorbidity Index. Material and social deprivation quintiles were based on the Québec Index of Material and Social Deprivation.^22^ Ethnicity was determined with a validated name recognition algorithm, Onomap. This algorithm assigned likely ethnicity based on name patterns and has been validated in previous studies.^23,24^ Categories included East Asian (individuals of Chinese, Japanese or Korean backgrounds), South Asian (individuals of Pakistani, Indian, Bangladeshi, Nepalese, or Sri Lankan backgrounds), and Other (including individuals of Black, Central/West Asian, Filipino, Indigenous, Latin American, Pacific Islander, Southeast Asian, White, and other ethnicities). We assessed health care use by the number of times any physician billing code or hospitalization code was recorded for each individual, considered as health care visits, from the date of first HCV diagnosis to last DAA treatment initiation (or the same date for matched untreated individual), and from 6 months post-DAA treatment to event or censor date. We categorized health care visits pretreatment and posttreatment using the 25th, 50th, and 75th percentiles based on the mean number of health care visits coded for overall study population. Detailed variable definitions can be found in eTable 2 in Supplement 1.

### Statistical Analysis

As the clinical and sociodemographic characteristics of people who received DAAs may differ from people who never received treatment, we used inverse probability of treatment weights (IPTW) to adjust for imbalances in baseline characteristics. IPTW were estimated for the average treatment effect (ATE) with propensity scores (eFigure 1 in Supplement 1).^25,26^ The balance across 3 groups—treated and SVR, treated and no SVR, and untreated—was assessed using the standardized mean difference (SMD) approach (eTable 4 in Supplement 1) and considered an SMD of less than 0.1 to indicate a balanced distribution.^27,28^

Study participants were followed up from the index date to the earliest of (1) diagnosis of EHM, (2) death, or (3) end of study (March 31, 2021). We generated cumulative incidence curves and computed incidence rates for each of the EHM groups. Then, we used IPTW-weighted Cox proportional cause-specific hazards multivariable regression model.^29^ Covariates were selected a priori based on literature as confounders of extrahepatic manifestations.^20^

We used cause-specific hazards models as our primary analysis approach, as our research question focused on the direct association between DAA treatment and the risk of developing specific extrahepatic manifestations. However, as a sensitivity analysis, we performed Fine-Gray subdistribution hazards models to account for the competing risk of death.

Furthermore, genotype 3 has been associated with a more aggressive natural history, higher risk of cirrhosis, and hepatocellular carcinoma.^30^ Older age is a major risk factor for several EHMs assessed in this analysis. For this reason, we performed subgroup analyses stratified by age (born on or before 1959, born after 1959), and for those identified with genotype 1 or genotype 3. Additionally, we conducted a sensitivity analysis excluding individuals with prior IFN-based treatment experience from the treated groups. This analysis was performed as previous literature suggested an association between IFN treatment and insulin resistance, which could potentially influence metabolic and related outcomes.^31^ Finally, we conducted an additional analysis comparing all treated individuals (regardless of SVR status) to untreated individuals.

We created the analytic dataset and produced plots using SAS Enterprise Guide version 8.3^32^ and performed statistical analyses in SAS and R version 4.3.3 (R Project for Statistical Computing).^33^ IPTW were estimated using the WeightIt package in R version 0.14.2 (R Project for Statistical Computing).^34^ Data were analyzed from February 2024 to March 2025.

## Results

### Study Participant Characteristics

The study included 22 576 individuals (mean [SD] age, 42.0 [12.0] years; 14 950 male [66.2%]): 10 237 never treated (ie, untreated), 11 953 treated and SVR, and 386 treated and no SVR. The median (IQR) age at first HCV diagnosis was 43 (35-51) years for the treated and SVR group and 40 (32-49) years for the untreated group. The treated and SVR group had 7785 males (65.1%), the treated and no SVR group had 281 males (72.8%), and the untreated group had 6884 males (67.3%). Individuals in the treated and SVR group were more likely to be born before 1964 (8494/11 953 [71.1%]) compared with the untreated group (5876/10 237 [57.4%]) and the treated and no SVR group (237/386 [61.4%]). The treated and no SVR group had higher pretreatment health care utilization (245/386 [63.5%] had more than 14 visits annually) compared with the treated and SVR group (6219/11 953 [52.0%]) and untreated group (4774/10 237 [46.6%]). Individuals in the untreated group had greater material and social deprivation, with 4091 individuals (40.0%) and 5400 individuals (52.7%) in the fifth quintile (ie, most deprived) for material and social deprivation, respectively (Table 1). Individuals in the treated and SVR group were more likely to have HCV genotype 1 (7837 of 11 953 [65.6%]) compared with untreated (4528 of 10 237 [44.2%]) and treated and no SVR group (221 of 386 [57.3%]) (Table 1).

**Table 1.** Baseline Characteristics of Study Participants Who Never Received Treatment and Those Treated With DAA (SVR, No SVR)

Covariate	Untreated (n = 10 237), No. (%)	Treated, No. (%)		*P* value^a^
		SVR (n = 11 953)	No SVR (n = 386)	
Sex				
Female	3353 (32.8)	4168 (34.9)	105 (27.5)	.03
Male	6884 (67.3)	7785 (65.1)	281 (72.8)	
Birth year				
<1945	653 (6.4)	412 (3.5)	17 (4.4)	<.001
1945-1964	5223 (51.0)	8082 (67.6)	220 (57.0)	
1965-1974	2342 (22.9)	2097 (17.5)	72 (18.7)	
≥1975	2019 (19.7)	1362 (11.4)	77 (20.0)	
Age at first HCV diagnosis, median (IQR), y	40 (32-49)	43 (35-51)	40 (31-50)	<.001
Annual average health care visits pretreatment^b^				
0-6	3369 (32.9)	2340 (19.6)	59 (15.3)	<.001
7-13	2094 (20.5)	3394 (28.4)	82 (21.2)	
14-27	2223 (21.7)	3341 (28.0)	117 (30.3)	
≥28	2551 (24.9)	2878 (24.1)	128 (33.2)	
Annual average health care visits posttreatment^c^				
0-2	3808 (37.2)	1662 (13.9)	74 (19.2)	.001
3-11	1961 (19.2)	3807 (31.8)	77 (19.9)	
12-31	1819 (17.8)	3677 (30.8)	97 (25.1)	
≥32	2649 (25.9)	2807 (23.5)	138 (35.8)	
Ethnicity^d^				
East Asian	249 (2.4)	367 (3.1)	8 (2.1)	.36
South Asian	294 (2.9)	385 (3.2)	8 (2.1)	
Other	9694 (94.7)	11 201 (93.7)	370 (95.9)	
Material deprivation, quintile				
First (most privileged)	1321 (12.9)	1754 (14.7)	49 (12.7)	<.001
Second	1241 (12.1)	1841 (15.4)	54 (14.0)	
Third	1400 (13.7)	2082 (17.4)	62 (16.1)	
Fourth	2115 (20.7)	2615 (21.9)	88 (22.8)	
Fifth (most deprived)	4091 (40.0)	3567 (29.8)	130 (33.7)	
Unknown	69 (0.7)	94 (0.8)	≤5	
Social deprivation, quintile				
First (most privileged)	754 (7.4)	1065 (8.9)	22 (5.7)	<.001
Second	1046 (10.2)	1502 (12.6)	44 (11.4)	
Third	1270 (12.4)	1910 (16.0)	63 (16.3)	
Fourth	1698 (16.6)	2194 (18.4)	58 (15.0)	
Fifth (most deprived)	5400 (52.7)	5188 (43.4)	196 (50.8)	
Unknown	69 (0.7)	94 (0.8)	≤5	
HCV genotype				
Genotype 1	4528 (44.2)	7837 (65.6)	221 (57.3)	<.001
Other^e^	5709 (55.8)	4116 (34.4)	165 (42.7)	
HBV infection	588 (5.7)	932 (7.8)	32 (8.3)	<.001
HIV infection	501 (4.9)	957 (8.0)	48 (12.4)	<.001
Hypertension	2556 (25.0)	3942 (33.0)	118 (30.6)	<.001
Statin use	1170 (11.4)	1636 (13.7)	42 (10.9)	<.001
Obesity	270 (2.6)	429 (3.6)	15 (3.9)	<.001
NAFLD	43 (0.4)	129 (1.1)	≤5	<.001
Cirrhosis	847 (8.3)	1871 (15.7)	92 (23.8)	<.001
Major mental illness	4399 (43.0)	4488 (37.5)	186 (48.2)	<.001
Alcohol use disorder	4086 (39.9)	3868 (32.4)	156 (40.4)	<.001
Drug dependence disorder	6632 (64.8)	5803 (48.5)	240 (62.2)	<.001
Injection drug use	5435 (53.1)	4572 (38.2)	204 (52.8)	<.001
Opioid agonist therapy	3930 (38.4)	3308 (27.7)	160 (41.5)	<.001
Elixhauser Comorbidity Index				
0	3187 (31.1)	4043 (33.8)	76 (19.7)	<.001
1	1851 (18.1)	2443 (20.4)	77 (19.9)	
≥2	5199 (50.8)	5467 (45.7)	233 (60.4)	

Abbreviations: DAA, direct-acting antivirals; HBV, hepatitis B virus; HCV, hepatitis C virus; HIV, human immunodeficiency virus; NAFLD, nonalcoholic fatty liver disease; Q, quintiles; SVR, sustained virologic response.

^a^ Mantel-Haenszel test (categorical variables) or Wilcoxon rank sum test (continuous variable) were used for comparison between study participants.

^b^ Health care visits pretreatment were assessed with number of times any physician billing code from Medical Services Plan or hospitalization code from Discharge Abstract Database was recorded for each individual from the date of first HCV diagnosis to last DAA treatment initiation (or the same date for matched untreated individual). Categories were created using 25th, 50th, and 75th percentiles based on average number of health care visits coded for the overall study population.

^c^ Health care visits posttreatment were assessed with number of times any physician billing code from Medical Services Plan or hospitalization code from Discharge Abstract Database was recorded for each individual from 6 months post-end of last DAA treatment (or the same date for matched untreated individual) to event or censor date. Categories were created using 25th, 50th, and 75th percentiles based on average number of health care visits coded for the overall study population.

^d^ East Asian category included individuals of Chinese, Japanese, or Korean backgrounds; South Asian category included individuals of Pakistani, Indian, Bangladeshi, Nepalese, or Sri Lankan backgrounds; Other category included residents of British Columbia with ethnic ancestry defined as White, Indigenous, Black, Latin American, Pacific Islander, Central/West Asian, Filipino, Southeast Asian, or Other.

^e^ Other category included HCV genotypes 2, 3, other, and unknown.

### Prevalence and Incidence of EHMs

In the overall study population, T2D was the most prevalent EHM, followed by MACE and CKD and ESKD, then neurocognitive disorders. The prevalence of stroke was the lowest among the assessed extrahepatic manifestations (eTable 3 in Supplement 1). Among older individuals (born on or before 1959), prevalence of EHMs was greater among those who never received treatment compared with those who were treated, except for T2D. Among younger individuals (born after 1959), EHM prevalence was greater among those who were treated. When looking at individuals identified with HCV genotype 1 compared with those identified with genotype 3, the prevalence of EHMs overall was similar, with individuals with genotype 1 having slightly higher prevalence of diabetes and MACE. There were no statistically significant differences in EHM prevalence among those who achieved SVR and those who did not.

**Figure 2.**
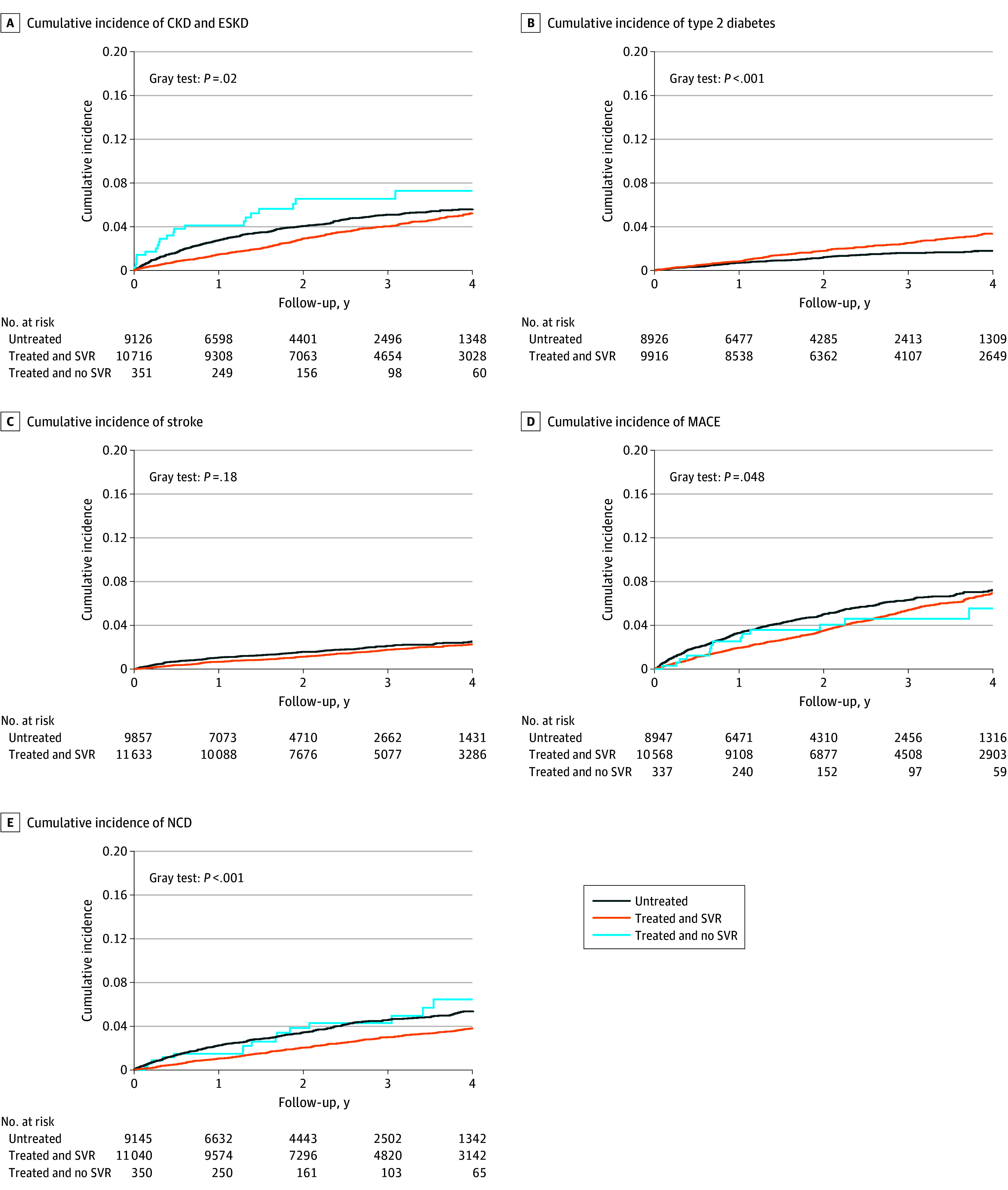
Cumulative Incidence of Extrahepatic Manifestations Between Individuals Who Never Received Treatment and Those Who Received Direct-Acting Antivirals Treatment Treated and no SVR group was excluded for T2D (panel B) and stroke (panel C), as the incident cases in this group were less than 5. CKD indicates chronic kidney disease; ESKD, end-stage kidney disease; MACE, major adverse cardiac events; NCD, neurocognitive disorders; T2D, type 2 diabetes.

As for the incident cases of EHMs, among the overall study population, individuals in the untreated group and those in the treated and no SVR group had higher IRs of EHMs compared with those who were in the treated and SVR group, except for T2D (Table 2). The incidence rates (per 1000 person-years) in untreated vs treated with SVR groups were: 21.0 (95% CI, 19.0-23.1) vs 14.7 (95% CI, 13.4-16.1) for CKD and ESKD, 8.9 (95% CI, 7.7-10.2) vs 6.3 (95% CI, 5.5-7.2) for stroke, 26.7 (95% CI, 24.5- 29.1) vs 19.3 (95% CI, 17.8-20.9) for MACE, 19.2 (95% CI, 17.3-21.2) vs 10.3 (95% CI, 9.2-11.5) for NCD, and 6.4 (95% CI, 5.4-7.7) vs 9.2 (95% CI, 8.1-10.4) for T2D. When stratified by age, older individuals had significantly higher IRs of EHMs compared with younger individuals. In the older group, IRs of EHMs were higher among those who were in the untreated group and in the treated and no SVR group compared with the treated and SVR group, including T2D; however, the 95% CIs overlapped. Among the younger group, while individuals who were untreated had higher IRs of EHMs compared with those who were treated and SVR, the 95% CIs overlapped for all EHMs, except T2D, where IR was significantly higher for treated and SVR compared with untreated. For individuals with HCV genotype 1, individuals in the untreated group had significantly higher IRs of EHMs, except for T2D. For genotype 3, those in the treated and SVR group had the highest IR of T2D, while those in the treated and no SVR group had the highest IR of CKD and ESKD. Figure 2 shows the cumulative incidence curves for each of the EHM groups.

**Table 2.** Incidence Rates (IRs) of Extrahepatic Manifestations Among Study Participants by DAA Treatment, Overall, Stratified by Age and Genotype

Outcome	IR (95% CI), per 1000 person-years		
	Untreated	Treated and SVR	Treated and no SVR
**Overall study population**			
CKD and ESKD	21.0 (19.0-23.1)	14.7 (13.4-16.1)	28.0 (18.2-42.9)
T2D	6.4 (5.4-7.7)	9.2 (8.1-10.4)	Not shown^a^
Stroke	8.9 (7.7-10.2)	6.3 (5.5-7.2)	Not shown^a^
MACE	26.7 (24.5-29.1)	19.3 (17.8-20.9)	20.7 (12.5-34.4)
NCD	19.2 (17.3-21.2)	10.3 (9.2-11.5)	19.5 (11.8-32.4)
**Born on or before 1959**			
CKD and ESKD	35.4 (31.3-40.0)	17.2 (15.4-19.2)	46.3 (28.8-74.4)
T2D	9.9 (7.7-12.6)	8.6 (7.2-10.1)	Not shown^a^
Stroke	15.1 (12.6-18.1)	7.8 (6.6-9.1)	Not shown^a^
MACE	48.2 (43.2-53.8)	24.1 (21.8-26.5)	38.5 (22.4-66.4)
NCD	34.7 (30.6-39.3)	12.5 (11.0-14.2)	28.8 (15.9-52.0)
**Born after 1959**			
CKD and ESKD	12.5 (10.7-14.7)	11.2 (9.5-13.2)	Not shown^a^
T2D	4.6 (3.6-6.0)	9.9 (8.3-11.9)	Not shown^a^
Stroke	5.1 (4.0-6.5)	4.2 (3.3-5.5)	Not shown^a^
MACE	15.5 (13.4-17.8)	13.1 (11.3-15.2)	Not shown^a^
NCD	10.3 (8.6-12.2)	7.1 (5.8-8.7)	Not shown^a^
**HCV GT1**			
CKD and ESKD	20.2 (17.4-23.5)	14.4 (12.9-16.1)	17.0 (8.5-34.1)
T2D	6.9 (5.3-8.9)	7.8 (6.7-9.2)	Not shown^a^
Stroke	9.0 (7.3-11.2)	5.9 (5.0-7.0)	Not shown^a^
MACE	27.1 (23.8-30.9)	18.9 (17.1-20.8)	18.0 (9.0-36.1)
NCD	19.0 (16.3-22.2)	9.8 (8.6-11.2)	19.0 (9.9-36.5)
**HCV GT3**			
CKD and ESKD	19.7 (15.6-24.8)	15.5 (12.5-19.3)	61.2 (34.7-107.7)
T2D	6.4 (4.3-9.7)	12.5 (9.7-16.0)	Not shown^a^
Stroke	6.0 (4.3-9.7)	6.4 (4.7-8.9)	Not shown^a^
MACE	22.0 (17.7-27.4)	18.2 (14.9-22.2)	29.8 (13.4-66.4)
NCD	14.7 (11.2-19.2)	11.3 (8.8-14.5)	24.5 (10.2-58.8)

Abbreviations: CKD, chronic kidney disease; DAA, direct-acting antivirals; ESKD, end-stage kidney disease; MACE, major adverse cardiac events; NCD, neurocognitive disorders; SVR, sustained virologic response; T2D, type 2 diabetes.

^a^ IRs are not shown where incident cases are less than 5.

### Association Between DAA and the Risk of EHMs

After adjusting for confounders, successful HCV treatment with DAA was associated with lower risk of incident CKD and ESKD (adjusted cause-specific hazard ratio [acsHR], 0.54; 95% CI, 0.47-0.63), stroke (acsHR, 0.66; 95% CI, 0.54-0.81), MACE (acsHR, 0.58; 95% CI, 0.52-0.66), and NCD (acsHR, 0.52; 95% CI, 0.45-0.66) compared with no treatment. T2D risk was not reduced (acsHR, 1.04; 95% CI, 0.84-1.29) (Table 3).

**Table 3.** Adjusted Cause-Specific Hazard Ratios for the Association Between Direct-Acting Antivirals and the Risk of Incident Extrahepatic Manifestations, Overall, Stratified by Age and Genotype

Outcomes	acsHR, (95% CI)^a^				
	Overall study population	Born on or before 1959	Born after 1959	HCV genotype 1	HCV genotype 3
**CKD and ESKD**					
Untreated	1 [Reference]	1 [Reference]	1 [Reference]	1 [Reference]	1 [Reference]
Treated and SVR	0.54 (0.47-0.63)	0.54 (0.45-0.65)	0.53 (0.41-0.68)	0.55 (0.45-0.67)	0.47 (0.33-0.66)
Treated and no SVR	0.43 (0.19-1.00)	0.58 (0.24-1.43)	NA	0.37 (0.12-1.17)	1.15 (0.36-2.89)
**T2D**					
Untreated	1 [Reference]	1 [Reference]	1 [Reference]	1 [Reference]	1 [Reference]
Treated and SVR	1.04 (0.84-1.29)	0.81 (0.60-1.09)	1.36 (0.99-1.88)	0.89 (0.66-1.22)	1.16 (0.72-1.86)
Treated and no SVR	NA	NA	NA	NA	NA
**Stroke**					
Untreated	1 [Reference]	1 [Reference]	1 [Reference]	1 [Reference]	1 [Reference]
Treated and SVR	0.66 (0.54-0.81)	0.66 (0.51-0.85)	0.61 (0.42-0.89)	0.62 (0.46-0.82)	0.87 (0.52-1.46)
Treated and no SVR	NA	NA	NA	NA	NA
**MACE**					
Untreated	1 [Reference]	1 [Reference]	1 [Reference]	1 [Reference]	1 [Reference]
Treated and SVR	0.58 (0.52-0.66)	0.59 (0.50-0.68)	0.54 (0.43-0.67)	0.58 (0.49-0.68)	0.64 (0.47-0.86)
Treated and no SVR	0.44 (0.21-0.89)	0.66 (0.32-1.36)	NA	0.42 (0.16-1.08)	0.59 (0.17-2.14)
**NCD**					
Untreated	1 [Reference]	1 [Reference]	1 [Reference]	1 [Reference]	1 [Reference]
Treated and SVR	0.52 (0.45-0.61)	0.50 (0.41-0.60)	0.51 (0.38-0.67)	0.49 (0.39-0.60)	0.64 (0.44-0.92)
Treated and no SVR	0.69 (0.34-1.40)	0.83 (0.37-1.88)	NA	0.59 (0.22-1.57)	0.75 (0.20-2.85)

Abbreviations: acsHR, adjusted cause-specific hazard ratio; CKD, chronic kidney disease; ESKD, end-stage kidney disease; MACE, major adverse cardiac events; NA, not applicable; NCD, neurocognitive disorders; SVR, sustained virologic response; T2D, diabetes.

^a^ Adjusted cause-specific hazard ratios were obtained from inverse probability of treatment weights for average treatment effect weighted cause-specific hazards models adjusted for sex (male, female), birth year, age at first hepatitis C diagnosis (years), ethnicity (East Asian, South Asian, Other), material deprivation quintiles, social deprivation quintiles, hepatitis C genotype (genotype 1, other), baseline diagnosis of hepatitis B infection, HIV infection, hypertension, statin use, obesity, nonalcoholic fatty liver disease, prevalent T2D (except in model for incident T2D), cirrhosis, major mental illness, alcohol use disorder, drug dependence disorder, injection drug use, and opioid agonist therapy.

These trends were consistent across age groups and HCV genotypes, except for stroke in genotype 3. Sensitivity analyses using Fine-Gray models showed similar trends, except for T2D (increased risk: adjusted subdistributional hazard ratio [asHR], 1.29; 95% CI, 1.04-1.60) and stroke (nonsignificant risk reduction: asHR, 0.86; 95% CI, 0.76-1.06) (eTable 5 in Supplement 1).

In a sensitivity analysis, excluding 1733 individuals with prior IFN experience from the treated groups, results remained consistent with our main findings (eTable 6 in Supplement 1). Successful DAA treatment remained significantly associated with lower risk of CKD and ESKD, stroke, MACE, and neurocognitive disorders compared with no treatment, with similar effect sizes to our primary analysis. The lack of association with T2D risk was also consistent in this sensitivity analysis.

When comparing all treated individuals (combined SVR and no SVR groups) vs untreated individuals, results were consistent with our main findings. The adjusted hazard ratios for all treated individuals compared with untreated were similar to those observed when comparing treated and SVR with untreated for all EHMs (eTable 7 in Supplement 1).

## Discussion

This large population-based cohort study from British Columbia, Canada, examined the association between successful HCV treatment with DAAs and EHMs among over 20 000 individuals with chronic HCV. After adjusting for baseline characteristics and potential confounders, successful DAA treatment was associated with lower risk of CKD, ESKD, hospitalized stroke, MACE, and NCD. However, DAA treatment was not associated with reduced risk of T2D; in fact, a higher incidence rate of T2D was observed among those who achieved SVR, particularly in individuals born after 1959 and those with HCV genotype 3. These findings demonstrate the potential of DAA treatment in reducing risk of multiple EHMs, while highlighting the need for further investigation into its association with T2D risk.

Consistent with our findings, previous studies have shown that DAA treatment is associated with lower risk of cardiovascular events. A US-based study among veterans found a 43% decreased risk of cardiovascular disease events in DAA-treated patients compared with untreated patients.^16^ Similarly, a French study^35^ reported decreased risk of peripheral arterial events and, in patients with advanced fibrosis, reduced risk of heart failure and overall cardiovascular events. Other research has linked HCV clearance by DAA treatment to decreased stroke and transient ischemic attack.^36^ Our study extends these findings to the general population diagnosed with HCV in Canada, demonstrating lower risk of ischemic and hemorrhagic stroke, TIA, and overall major adverse cardiac events associated with successful DAA treatment. The large sample size of our study allowed for more precise estimates of incidence and meaningful comparisons between treated and untreated groups.

In our study, we found that successful HCV treatment with DAAs was not significantly associated with reduced risk of diabetes compared with no treatment. There may be a few potential reasons behind this result. It is possible that people who received DAA treatment had improved access to health care and services compared with those who were never treated. In our study, while we attempted to adjust for the differences in health care use by including the annual average health care visits pre- and posttreatment, these were based on physician billing or hospitalization codes and may be missing some nuances, such as integrated health care or attachment to a family physician. It may be that people who were treated for HCV had better quality of access to ongoing health care and screening, leading to higher frequency of being diagnosed with T2D compared with those who were never treated for HCV. Limited evidence exists regarding the association between DAAs and incident diabetes. Adinolfi et al^37^ found in a prospective study based in a clinical setting that successful treatment with DAAs was associated with significant reduction in the risk of developing diabetes compared with no treatment, in both F0 to F2 and F3 to F4 fibrosis groups. Other studies showed that excess weight gain is common following DAA treatment,^38,39^ and the worsening of the systemic metabolic profile associated with weight gain may explain weakened clinical response related to hepatic outcomes post-SVR.^40^ It is possible that this metabolic profile change post-SVR also interferes with the impact of DAA treatment on the development of diabetes.

While few studies have investigated the association between IFN-free DAAs and kidney diseases and kidney function, SVR has been linked to reduced risk of chronic kidney diseases and end-stage kidney disease.^41,42^ One study found that DAA treatment stabilized or improved estimated glomerular filtration rate, particularly in patients without diabetes.^43^ Regarding neurocognitive disorders, although chronic HCV has been suggested as a risk factor,^44,45^ research on associations with DAA treatment is limited. A small study of 23 participants reported improvements in cognitive functions following SVR achieved through DAA treatment.^46^ Our findings extend this research, demonstrating that successful DAA treatment was associated with reduced risk of both chronic kidney and end-stage kidney diseases, as well as neurocognitive disorders, including dementia, delirium, and Alzheimer disease.

### Strengths and Limitations

A major strength of our study was the availability of large, population-based data, which allowed for the assessment of DAA treatment among over 22 000 people in British Columbia. The large sample size enabled the assessment of multiple extrahepatic outcomes. The comprehensive data also allowed for multivariable analysis adjusting for important confounders. The median follow-up time in our study was about 2.5 years (after SVR assessment), which allowed for a longer period of follow-up for outcome assessment.

However, this study has limitations. The use of administrative health data may lead to outcome misclassification, although this is likely nondifferential between treated and untreated groups, potentially biasing estimates toward the null.

A critical limitation is the difference in baseline characteristics between treated and untreated individuals, stemming from the phased roll out of DAAs in British Columbia between 2014 and 2018. This introduces confounding by indication, as treated individuals may have a higher a priori risk of EHMs due to advanced fibrosis and other clinical characteristics. Indeed, baseline differences were observed for HIV and HBV coinfection, hypertension, statin use, obesity, nonalcoholic fatty liver disease, and cirrhosis (eTable 4 in Supplement 1).

To address these imbalances, we used IPTW and double adjustment in regression models.^47,48^ However, residual confounding may remain. The administrative data lacks some clinical details that could influence EHM risk assessment, including baseline kidney function for kidney outcomes, aspirin use for cardiovascular outcomes, and newer antidiabetic medications like glucagon-like peptide-1 (ie, GLP-1) receptor agonists and sodium-glucose cotransporter 2 (ie, SGLT2) inhibitors for diabetic outcomes, potentially affecting the precision of the estimates. However, subgroup analyses and sensitivity analyses showed consistent beneficial effects of DAA treatment on EHMs. We used both cause-specific and subdistribution hazards models to provide complementary perspectives on the association between DAA treatment and EHMs. The cause-specific models were chosen for primary analysis due to their focus on assessing the direct association between DAA treatment and incident EHMs, while the subdistribution models accounted for the competing risk of death, which is particularly relevant in this population. The consistency of findings across both analytical approaches strengthens our confidence in the observed associations.^49,50^

In addition, while our median follow-up of 2.5 years after SVR assessment may be considered relatively short for some chronic outcomes, it was sufficient to observe a meaningful number of EHM events across all study groups. Our large sample size of over 22 000 individuals provided adequate statistical power to detect significant associations within this time frame. Nevertheless, longer follow-up would be valuable to further understand the long-term associations between DAA treatment and EHMs, particularly for outcomes that may take longer to develop.

Finally, the definition of incident EHMs in this study included all new diagnoses occurring after the index date. A more conservative landmark approach that excludes events occurring within 6 to 12 months post index date could potentially provide additional insights into the temporal association between HCV clearance and extrahepatic benefits by allowing more time for biological effects to manifest and reducing potential detection bias. However, we believe that the adjustment for health care use patterns and sufficient median follow-up time likely provided adequate control for such potential bias in this analysis.

## Conclusions

This large population-based cohort study in British Columbia suggested that successful HCV treatment with DAAs was associated with lower risk of multiple extrahepatic manifestations. Recent studies^51,52,53,54^ suggest that extrahepatic damage may persist after HCV elimination, especially in patients with comorbidities like cirrhosis or diabetes, underscoring the need for early screening and prompt HCV treatment. The findings of this study further emphasize the urgency of addressing the substantial gap in treatment uptake. Despite the availability of highly effective therapies, approximately half of individuals diagnosed with HCV in our cohort remained untreated by 2020, reflecting the persistent challenges in linkage-to-care that have been identified as critical barriers to HCV elimination.^55^ The potential extrahepatic benefits of HCV treatment highlighted in this study provide additional rationale for enhanced efforts to identify and overcome barriers to care, including reducing stigma, increasing clinician awareness, addressing socioeconomic challenges, and implementing innovative care models to improve overall health of people affected by HCV.

## Notes

Supplement 1. **eTable 1. **Criteria and data sources for the BC Hepatitis Testers Cohort (BC-HTC) **eTable 2. **Case definitions of variables used in the analysis **eTable 3.** Prevalence of extrahepatic manifestations at baseline among study participants by treatment status, overall, stratified by age and genotype **eTable 4.** Comparison of baseline characteristics between untreated, treated and SVR, and treated and no SVR groups in the study population and in the inverse probability of treatment weights (IPTW)-weighted dataset **eTable 5. **Adjusted subdistributional hazard ratios for the effect of direct-acting antivirals and sustained virologic response (SVR) on the risk of incident extrahepatic manifestations, overall and stratified by age **eTable 6.** Adjusted cause-specific and subdistributional hazard ratios for the association between direct-acting antivirals and the risk of incident extrahepatic manifestations, in the overall study population excluding IFN-experienced individuals **eTable 7. **Adjusted cause-specific and subdistributional hazard ratios for the association between direct-acting antivirals and the risk of incident extrahepatic manifestations, in the overall study population comparing treated vs untreated groups **eFigure.** Propensity score density plot of overall study population

Supplement 2.
Data Sharing Statement

## References

[1] Global hepatitis report, 2017. World Health Organization. Accessed May 7, 2025. https://www.who.int/publications/i/item/9789241565455

[2] Negro F, Forton D, Craxì A, Sulkowski MS, Feld JJ, Manns MP. Extrahepatic morbidity and mortality of chronic hepatitis C. Gastroenterology. 2015;149(6):1345-1360. doi:10.1053/j.gastro.2015.08.035 26319013

[3] Saadoun D, Landau DA, Calabrese LH, Cacoub PP. Hepatitis C-associated mixed cryoglobulinaemia: a crossroad between autoimmunity and lymphoproliferation. Rheumatology (Oxford). 2007;46(8):1234-1242. doi:10.1093/rheumatology/kem132 17566058

[4] Cacoub P, Desbois AC, Isnard-Bagnis C, Rocatello D, Ferri C. Hepatitis C virus infection and chronic kidney disease: time for reappraisal. 2016;65(1):S82-S94. doi:10.1016/j.jhep.2016.06.011 27641990

[5] Pol S, Lagaye S. The remarkable history of the hepatitis C virus. Genes Immun. 2019;20(5):436-446. doi:10.1038/s41435-019-0066-z 31019253

[6] Negro F. Natural history of hepatic and extrahepatic hepatitis C virus diseases and impact of interferon-free hcv therapy. Cold Spring Harb Perspect Med. 2020;10(4):a036921. doi:10.1101/cshperspect.a036921 31636094 PMC7117949

[7] Kohli A, Shaffer A, Sherman A, Kottilil S. Treatment of hepatitis C: a systematic review. JAMA. 2014;312(6):631-640. doi:10.1001/jama.2014.7085 25117132

[8] Nahon P, Bourcier V, Layese R, ; ANRS CO12 CirVir Group. Eradication of hepatitis C virus infection in patients with cirrhosis reduces risk of liver and non-liver complications. Gastroenterology. 2017;152(1):142-156.e2. doi:10.1053/j.gastro.2016.09.009 27641509

[9] Mahale P, Engels EA, Li R, . The effect of sustained virological response on the risk of extrahepatic manifestations of hepatitis C virus infection. Gut. 2018;67(3):553-561. doi:10.1136/gutjnl-2017-313983 28634198 PMC6292199

[10] Hsu YCY, Ho HJ, Huang YT, . Association between antiviral treatment and extrahepatic outcomes in patients with hepatitis C virus infection. Gut. 2015;64(3):495-503. doi:10.1136/gutjnl-2014-308163 25398770

[11] Rossi C, Jeong D, Wong S, ; BC Hepatitis Testers Cohort Team. Sustained virological response from interferon-based hepatitis C regimens is associated with reduced risk of extrahepatic manifestations. J Hepatol. 2019;71(6):1116-1125. doi:10.1016/j.jhep.2019.07.021 31433302

[12] Marshall AD, Saeed S, Barrett L, ; Canadian Network on Hepatitis C (CanHepC). Restrictions for reimbursement of direct-acting antiviral treatment for hepatitis C virus infection in Canada: a descriptive study. CMAJ Open. 2016;4(4):E605-E614. doi:10.9778/cmajo.20160008 28018873 PMC5173474

[13] Chronic Hepatitis C Medication Now Available for All British Columbians. British Columbia Ministry of Health. Accessed May 7, 2025. https://news.gov.bc.ca/releases/2018HLTH0017-000387

[14] Mohanty A, Salameh S, Butt AA. Impact of direct acting antiviral agent therapy upon extrahepatic manifestations of hepatitis C virus infection. Curr HIV/AIDS Rep. 2019;16(5):389-394. doi:10.1007/s11904-019-00466-1 31482299

[15] Gastaldi G, Gomes D, Schneiter P, . Treatment with direct-acting antivirals improves peripheral insulin sensitivity in non-diabetic, lean chronic hepatitis C patients. PLoS One. 2019;14(6):e0217751. doi:10.1371/journal.pone.0217751 31170218 PMC6553748

[16] Butt AA, Yan P, Shuaib A, Abou-Samra AB, Shaikh OS, Freiberg MS. Direct-acting antiviral therapy for HCV infection is associated with a reduced risk of cardiovascular disease events. Gastroenterology. 2019;156(4):987-996.e8. doi:10.1053/j.gastro.2018.11.022 30445009

[17] Liu CH, Lin JW, Liu CJ, . Long-term evolution of estimated glomerular filtration rate in patients with antiviral treatment for hepatitis C virus infection. Clin Gastroenterol Hepatol. 2023;21(2):424-434.e5. doi:10.1016/j.cgh.2022.01.050 35131346

[18] Janjua NZ, Kuo M, Chong M, . Assessing hepatitis C burden and treatment effectiveness through the British Columbia hepatitis testers cohort (BC-HTC): design and characteristics of linked and unlinked participants. PLoS One. 2016;11(3):e0150176. doi:10.1371/journal.pone.0150176 26954020 PMC4783072

[19] Karim ME, Gustafson P, Petkau J, Tremlett H; Long-Term Benefits and Adverse Effects of Beta-Interferon for Multiple Sclerosis (BeAMS) Study Group. Comparison of statistical approaches for dealing with immortal time bias in drug effectiveness studies. Am J Epidemiol. 2016;184(4):325-335. doi:10.1093/aje/kwv445 27455963 PMC4983651

[20] Cacoub P, Saadoun D. Extrahepatic manifestations of chronic HCV infection. N Engl J Med. 2021;384(11):1038-1052. doi:10.1056/NEJMra2033539 33730456

[21] Mazzaro C, Quartuccio L, Adinolfi LE, . A review on extrahepatic manifestations of chronic hepatitis C virus infection and the impact of direct-acting antiviral therapy. Viruses. 2021;13(11):2249. doi:10.3390/v13112249 34835054 PMC8619859

[22] Pampalon R, Gamache P, Hamel D. The Québec Index of material and social deprivation: methodological follow-up, 1991 through 2006. Institut national de santé publique du Québec. Accessed May 7, 2025. https://www.inspq.qc.ca/en/publications/1258

[23] Lakha F, Gorman DR, Mateos P. Name analysis to classify populations by ethnicity in public health: validation of Onomap in Scotland. Public Health. 2011;125(10):688-696. doi:10.1016/j.puhe.2011.05.003 21907365

[24] Ryan R, Vernon S, Lawrence G, Wilson S. Use of name recognition software, census data and multiple imputation to predict missing data on ethnicity: application to cancer registry records. BMC Med Inform Decis Mak. 2012;12:3. doi:10.1186/1472-6947-12-3 22269985 PMC3353229

[25] Desai RJ, Franklin JM. Alternative approaches for confounding adjustment in observational studies using weighting based on the propensity score: a primer for practitioners. BMJ. 2019;367:l5657. doi:10.1136/bmj.l5657 31645336

[26] Austin PC, Stuart EA. Moving towards best practice when using inverse probability of treatment weighting (IPTW) using the propensity score to estimate causal treatment effects in observational studies. Stat Med. 2015;34(28):3661-3679. doi:10.1002/sim.6607 26238958 PMC4626409

[27] Austin PC. Balance diagnostics for comparing the distribution of baseline covariates between treatment groups in propensity-score matched samples. Stat Med. 2009;28(25):3083-3107. doi:10.1002/sim.3697 19757444 PMC3472075

[28] Zhang Z, Kim HJJ, Lonjon G, Zhu Y; written on behalf of AME Big-Data Clinical Trial Collaborative Group. Balance diagnostics after propensity score matching. Ann Transl Med. 2019;7(1):16-16. doi:10.21037/atm.2018.12.10 30788363 PMC6351359

[29] Lau B, Cole SR, Gange SJ. Competing risk regression models for epidemiologic data. Am J Epidemiol. 2009;170(2):244-256. doi:10.1093/aje/kwp107 19494242 PMC2732996

[30] Kanwal F, Kramer JR, Ilyas J, Duan Z, El-Serag HB. HCV genotype 3 is associated with an increased risk of cirrhosis and hepatocellular cancer in a national sample of US veterans with HCV. Hepatology. 2014;60(1):98-105. doi:10.1002/hep.27095 24615981 PMC4689301

[31] Dai CY, Huang JF, Hsieh MY, . Insulin resistance predicts response to peginterferon-alpha/ribavirin combination therapy in chronic hepatitis C patients. J Hepatol. 2009;50(4):712-718. doi:10.1016/j.jhep.2008.12.017 19231011

[32] SAS. SAS Institute Inc. Accessed May 7, 2025. https://www.sas.com/en_us/home.html

[33] R: A language and environment for statistical computing. R Core Team. Accessed May 7, 2025. https://www.r-project.org

[34] Greifer N. WeightIt: weighting for covariate balance in observational studies. CRAN-R Project. Accessed May 7, 2025. https://CRAN.R-project.org/package=WeightIt

[35] Lam L, Fontaine H, Lapidus N, ; ANRS/AFEF Hepather study group. Impact of direct-acting antiviral treatment for hepatitis C on cardiovascular diseases and extrahepatic cancers. Pharmacoepidemiol Drug Saf. 2023;32(4):486-495. doi:10.1002/pds.5576 36444965

[36] Roguljic H, Nincevic V, Bojanic K, . Impact of DAA treatment on cardiovascular disease risk in chronic HCV infection: an update. Front Pharmacol. 2021;12:678546. doi:10.3389/fphar.2021.678546 34045969 PMC8144519

[37] Adinolfi LE, Petta S, Fracanzani AL, . Reduced incidence of type 2 diabetes in patients with chronic hepatitis C virus infection cleared by direct-acting antiviral therapy: a prospective study. Diabetes Obes Metab. 2020;22(12):2408-2416. doi:10.1111/dom.14168 32761721

[38] Do A, Esserman DA, Krishnan S, . Excess weight gain after cure of hepatitis C infection with direct-acting antivirals. J Gen Intern Med. 2020;35(7):2025-2034. doi:10.1007/s11606-020-05782-6 32342483 PMC7352003

[39] Schlevogt B, Boeker KHW, Mauss S, . Weight gain after interferon-free treatment of chronic hepatitis C-Results from the german hepatitis C-registry (DHC-R). Biomedicines. 2021;9(10):1495. doi:10.3390/biomedicines9101495 34680612 PMC8533115

[40] Leslie J, Geh D, Elsharkawy AM, Mann DA, Vacca M. Metabolic dysfunction and cancer in HCV: shared pathways and mutual interactions. J Hepatol. 2022;77(1):219-236. doi:10.1016/j.jhep.2022.01.029 35157957

[41] Aby ES, Dong TS, Kawamoto J, Pisegna JR, Benhammou JN. Impact of sustained virologic response on chronic kidney disease progression in hepatitis C. World J Hepatol. 2017;9(36):1352-1360. doi:10.4254/wjh.v9.i36.1352 29359019 PMC5756725

[42] Huang CF, Tseng KC, Cheng PN, . Impact of sofosbuvir-based direct-acting antivirals on renal function in chronic hepatitis C patients with impaired renal function: a large cohort study from the nationwide HCV Registry Program (TACR). Clin Gastroenterol Hepatol. 2022;20(5):1151-1162. doi:10.1016/j.cgh.2021.07.037 34333150

[43] Sise ME, Chute DF, Oppong Y, . Direct-acting antiviral therapy slows kidney function decline in patients with hepatitis C virus infection and chronic kidney disease. Kidney Int. 2020;97(1):193-201. doi:10.1016/j.kint.2019.04.030 31337501 PMC7094798

[44] Tsai HH, Liou HH, Muo CH, Lee CZ, Yen RF, Kao CH. Hepatitis C virus infection as a risk factor for Parkinson disease: a nationwide cohort study. Neurology. 2016;86(9):840-846. doi:10.1212/WNL.0000000000002307 26701382

[45] Wijarnpreecha K, Chesdachai S, Jaruvongvanich V, Ungprasert P. Hepatitis C virus infection and risk of Parkinson’s disease: a systematic review and meta-analysis. Eur J Gastroenterol Hepatol. 2018;30(1):9-13. doi:10.1097/MEG.0000000000000991 29049127

[46] Vaghi G, Gori B, Strigaro G, . Direct antivirals and cognitive impairment in hepatitis C: a clinical-neurophysiologic study. J Neurovirol. 2020;26(6):870-879. doi:10.1007/s13365-020-00904-6 32910431 PMC7716927

[47] Hernán MA, Robins JM. Using big data to emulate a target trial when a randomized trial is not available. Am J Epidemiol. 2016;183(8):758-764. doi:10.1093/aje/kwv254 26994063 PMC4832051

[48] Nguyen TL, Collins GS, Spence J, . Double-adjustment in propensity score matching analysis: choosing a threshold for considering residual imbalance. BMC Med Res Methodol. 2017;17(1):78. doi:10.1186/s12874-017-0338-0 28454568 PMC5408373

[49] Austin PC, Lee DS, Fine JP. Introduction to the analysis of survival data in the presence of competing risks. Circulation. 2016;133(6):601-609. doi:10.1161/CIRCULATIONAHA.115.017719 26858290 PMC4741409

[50] Wolbers M, Koller MT, Stel VS, . Competing risks analyses: objectives and approaches. Eur Heart J. 2014;35(42):2936-2941. doi:10.1093/eurheartj/ehu131 24711436 PMC4223609

[51] Sollima S, Milazzo L, Peri AM, Torre A, Antinori S, Galli M. Persistent mixed cryoglobulinaemia vasculitis despite hepatitis C virus eradication after interferon-free antiviral therapy. Rheumatology (Oxford). 2016;55(11):2084-2085. doi:10.1093/rheumatology/kew268 27338085

[52] Benhammou JN, Moon AM, Pisegna JR, . Nonalcoholic fatty liver disease risk factors affect liver-related outcomes after direct-acting antiviral treatment for hepatitis C. Dig Dis Sci. 2021;66(7):2394-2406. doi:10.1007/s10620-020-06457-2 32654086 PMC7854862

[53] Nakagawa M, Asahina Y, Kakinuma S, Okamoto R. Impact of eradication of hepatitis C virus on liver-related and -unrelated diseases: morbidity and mortality of chronic hepatitis C after SVR. J Gastroenterol. 2023;58(4):299-310. doi:10.1007/s00535-022-01940-1 36585501

[54] Witte M, Oltmanns C, Tauwaldt J, . The impact of cirrhosis on the inflammatory milieu before and long-term after hepatitis C virus elimination by direct-acting antiviral therapy. MedRXiv. Published online October 31, 2023. doi:10.1101/2023.10.31.23297828

[55] Yu ML, Ward JW. Sharing lessons learned to build effective hepatitis elimination programs. J Infect Dis. 2023;228(suppl 3):S145-S147. doi:10.1093/infdis/jiad342 37703338

