# Supplementary material for: Direct-Acting Antivirals and Risk of Hepatitis C Extrahepatic Manifestations
Source: JAMA Netw Open. 2025 Jun 11;8(6):e2514631. doi: 10.1001/jamanetworkopen.2025.14631 (PMC12159775; doi:10.1001/jamanetworkopen.2025.14631)
Supplement: Supplement 1. — eTable 1. Criteria and data sources for the BC Hepatitis Testers Cohort (BC-HTC) eTable 2. Case definitions of variables used in the analysis eTable 3. Prevalence of extrahepatic manifestations at baseline among study participants by treatment status, overall, stratified by age and genotype eTable 4. Comparison of baseline characteristics between untreated, treated and SVR, and treated and no SVR groups in the study population and in the inverse probability of treatment weights (IPTW)-weighted dataset eTable 5. Adjusted subdistributional hazard ratios for the effect of direct-acting antivirals and sustained virologic response (SVR) on the risk of incident extrahepatic manifestations, overall and stratified by age eTable 6. Adjusted cause-specific and subdistributional hazard ratios for the association between direct-acting antivirals and the risk of incident extrahepatic manifestations, in the overall study population excluding IFN-experienced individuals eTable 7. Adjusted cause-specific and subdistributional hazard ratios for the association between direct-acting antivirals and the risk of incident extrahepatic manifestations, in the overall study population comparing treated vs untreated groups eFigure. Propensity score density plot of overall study population [file jamanetwopen-e2514631-s001.pdf]

Supplemental Online Content

Jeong D, Wong S, Karim ME, et al. Direct-acting antivirals and risk of Hepatitis C extrahepatic manifestations. *JAMA Netw Open*. 2025;8(6):e2514631. doi:10.1001/jamanetworkopen.2025.14631

eTable 1. Criteria and data sources for the BC Hepatitis Testers Cohort (BC-HTC)

eTable 2. Case definitions of variables used in the analysis

eTable 3. Prevalence of extrahepatic manifestations at baseline among study participants by treatment status, overall, stratified by age and genotype

eTable 4. Comparison of baseline characteristics between untreated, treated and SVR, and treated and no SVR groups in the study population and in the inverse probability of treatment weights (IPTW)-weighted dataset

eTable 5. Adjusted subdistributional hazard ratios for the effect of direct-acting antivirals and sustained virologic response (SVR) on the risk of incident extrahepatic manifestations, overall and stratified by age

eTable 6. Adjusted cause-specific and subdistributional hazard ratios for the association between direct-acting antivirals and the risk of incident extrahepatic manifestations, in the overall study population excluding IFN-experienced individuals

eTable 7. Adjusted cause-specific and subdistributional hazard ratios for the association between direct-acting antivirals and the risk of incident extrahepatic manifestations, in the overall study population comparing treated vs untreated groups

eFigure 1. Propensity score density plot of overall study population

This supplemental material has been provided by the authors to give readers additional information about their work.

© 2025 Jeong D et al. *JAMA Network Open*.

eTable 1. Criteria and data sources for the BC Hepatitis Testers Cohort (BC-HTC)

Criteria for Inclusion in BC-HTC

All individuals:

- tested at the BCCDC Public Health Laboratory (BC-PHL) for HCV or HIV OR
- reported to BC public health as a confirmed case of HCV, HIV/AIDS, HBV or active TB OR
- included in BC Enhanced Strain Surveillance System (EHSSS) as an acute HBV or HCV case

All individuals meeting at least one the above criteria were linked internally across all their tests and case reports. Those with a valid personal health number (PHN) were then sent for deterministic linkage with the province-wide Cancer and Ministry of Health (MoH) datasets

**Provincial Communicable Disease Data Sources:**	**Data Date Ranges:**
BC-PHMRL HIV laboratory testing datasets (tests: ELISA, Western blot, NAAT, p24, culture)	1988–2015
BC-PHMRL HCV laboratory tests datasets (tests: antibody, HCV RNA, genotyping)	1992–2021, Oct
HIV/AIDS Information System (HAISYS) (public health HIV/AIDS case reports)	1980–2015
Integrated Public Health information System (iPHIS) (public health case reports of HCV, HBV, and TB)	1990–2015
Enhanced Strain Surveillance System (EHSSS) (risk factor data on a subset of acute HCV and acute HBV cases)	2000–2013
**Cancer and MoH Administrative Data Sources:**	**Data Date Ranges:**
Client Roster (CR) (Registry of enrollment in the universal public health insurance plan including residential history) [S1]	1990-2021, Mar
BC Cancer Registry (BCCR) (primary tumour registry, excludes metastatic cancers)	1923–2019
Discharge Abstracts Dataset (DAD) (hospitalization records) [S2]	1985–2021, Mar
Medical Services Plan (MSP) (physician diagnostic and billing data) [S3]	1990–2021, Mar
PharmaCare/PharmaNet (Pharma) (prescription drug dispensations) [S4, S5]	1985–2021, Dec
BC Vital Statistics (VS) (deaths registry) [S6]	1985–2021, Dec
NACRS (Emergency Departments) [S7]	2012–2021, Mar
Chronic Disease Registry [S8]	1992–2020, Mar

The final BC-HTC comprises all individuals successfully linked on PHN to the MoH Client Roster [S1] (a registry of all BC residents enrolled in the publicly-funded universal healthcare system)

Abbreviations: HCV, Hepatitis C Virus; HBV, Hepatitis B Virus; HIV/AIDS, Human Immunodeficiency Virus/Acquired Immunodeficiency Syndrome; BC-PHMRL, BC Public Health Microbiology and Reference Laboratory; RNA, Ribonucleic Acid; PCR, Polymerase Chain Reaction.

eReferences:

S1. British Columbia Ministry of Health [creator]. Client Roster (Client Registry System/Enterprise Master Patient Index). British Columbia Ministry of Health [publisher]. Data Extract. MOH (2013). 2021. https://www2.gov.bc.ca/gov/content/health/health-forms/online-services

S2. British Columbia Ministry of Health [creator]. Discharge Abstract Database (Hospital Separations). British Columbia Ministry of Health [publisher]. Data Extract. MOH (2013). 2021. https://www2.gov.bc.ca/gov/content/health/health-forms/online-services

S3. British Columbia Ministry of Health [creator]. Medical Services Plan (MSP) Payment Information File. British Columbia Ministry of Health [publisher]. Data Extract. MOH (2013). 2021.https://www2.gov.bc.ca/gov/content/health/health-forms/online-services

© 2025 Jeong D et al. *JAMA Network Open*.

S4. British Columbia Ministry of Health [creator]. PharmaCare. British Columbia Ministry of Health [publisher]. Data Extract. MOH (2013). 2021. https://www2.gov.bc.ca/gov/content/health/health-forms/online-services
S5. British Columbia Ministry of Health [creator]. PharmaNet. British Columbia Ministry of Health [publisher]. Data Extract. MOH (2013). 2021. https://www2.gov.bc.ca/gov/content/health/health-forms/online-services
S6. BC Vital Statistics Agency [creator]. Vital Statistics Deaths. BC Vital Statistics Agency [publisher]. Data Extract. BC Vital Statistics Agency (2014). 2021. https://www2.gov.bc.ca/gov/content/health/health-forms/online-services
S7. British Columbia Ministry of Health [creator]. National Ambulatory Care Reporting System. British Columbia Ministry of Health [publisher]. Data Extract. MOH (2017). 2021. https://www2.gov.bc.ca/gov/content/health/health-forms/online-services
S8. British Columbia Ministry of Health [creator]. Chronic Disease Registry. British Columbia Ministry of Health [publisher]. Data Extract. MOH. (2017) 2021. https://www2.gov.bc.ca/gov/content/health/health-forms/online-services

eTable 2. Case definitions of variables used in the analysis

**Alcohol use disorder**<br>Alcohol use disorder was defined at the first occurrence of two physician visits, or one hospitalization codes for major alcohol-related diagnoses including alcoholic mental disorders and dependence/abuse syndromes; alcoholic polyneuropathy, myopathy, cardiomyopathy; pseudo Cushing's syndrome; or discharge to alcohol rehabilitation, counselling, or surveillance.<br>**Physician Billing Data:** MSP ICD-9 diagnostic codes: starting with 291, 303, 3050, 3575, 4255<br>**Hospitalization Data:** DAD1/DAD3 ICD-9-CM diagnostic codes: starting with 291, 303, 3050, 3575, 4255; DAD2/NACRS/VS ICD-10-CA diagnostic codes: starting with F10, E244, G312, G621, G721, I426, Z502, Z714; exact K292, K700, K701, K702, K703, K704, K709, K852, K860, R780, X65, Y15
**Chronic kidney disease**<br>Chronic kidney disease was defined as one hospitalization or urgent care; or two physician visits in one year with ICD code(s) specified below. The case definition applies to persons aged 1 and older.<br>**Physician Billing Data:** MSP ICD-9 diagnostic codes: starting with '24940','24941','2504','28521','403','4040','4041','4042','4043','4044','4045','4046','4047','4048','40490','40491','40492','40493','581','582','583','584','585','586','587','7532','7534','7536','7537','7538','7539','V1303','V1304','V1305','V1306','V1307','V1308','V1309','V186','V420','V4510','V4511','V4512','V4573','V560','V561','V562','V563','V564','V565','V567','V568','V594','V815','V816'<br>**Hospitalization Data:** DAD1/DAD3/ICD-9-CM diagnostic codes: starting with '24940','24941','2504','28521','403','4040','4041','4042','4043','4044','4045','4046','4047','4048','40490','40491','40492','40493','581','582','583','584','585','586','587','7532','7534','7536','7537','7538','7539','V1303','V1304','V1305','V1306','V1307','V1308','V1309','V186','V420','V4510','V4511','V4512','V4573','V560','V561','V562','V563','V564','V565','V567','V568','V594','V815','V816'<br>DAD2/NACRS/VS ICD-10-CA diagnostic codes: starting with 'D631', 'E082', 'E102','E112', 'E122', 'E132', 'E142','I12','I13','N02','N03','N04','N04','N05','N06','N07','N080','N081','N082','N083','N084','N085','N086','N087','N088','N18','N19','Q620','Q621','Q623','Q624','Q625','Q626','Q627','Q628','Q642','Q643','Q644','Q645','Q646','Q647','Q648','Q649','Z490','Z491','Z492','Z4930','Z4931','Z4932','Z524','Z992'
**Cirrhosis**<br>Cirrhosis was defined with end-stage liver disease secondary to cirrhosis, at the first occurrence of either one physician visit or one hospitalization code relevant to decompensated cirrhosis (esophageal varices, SBP, hepatorenal syndrome, ascites, and portal hypertension) and compensated cirrhosis (chronic hepatitis failure (alcoholic/non-alcoholic), hepatic failure, unspecified, unspecified cirrhosis of the liver, other cirrhosis of the liver).<br>**Physician Billing Data:** MSP ICD-9 diagnostic codes: starting with 4562; exact codes: 4560, 4561, 5712, 5713, 5715, 5722, 5723, 5724, 5728, 7895, 56723, 07044<br>**Hospitalization Data:** DAD1/DAD3 ICD-9-CM diagnostic codes: starting with 4562; exact codes: 4560, 4561, 5712, 5713, 5715, 5722, 5723, 5724, 5728, 7895, 56723, 07044; DAD2/NACRS/VS ICD-10-CA

diagnostic codes: starting with K703; exact codes: I850, I982, I983, K652, K704, K721, K729, K7460, K7469, K766, K767, R18, I859, K717; CCI procedure codes: exact codes 1NA13BAFA, 1NA13BAX7, 1NA13BABD, 1KO76GPNR, 1OT52HA
**Direct Acting Antiviral (DAA) Treatments:** Following treatments were available in BC and prescribed to individuals in the study. sofosbuvir/ribavirin, sofosbuvir/ledipasvir, sofosbuvir/velpatasvir, sofosbuvir/velpatasvir/voxilaprevir, sofosbuvir + simeprevir, sofosbuvir + daclatasvir, sofosbuvir + boceprevir, paritaprevir/ritonavir/ombitasvir/dasabuvir, glecaprevir/pibrentasvir, and elbasvir/grazoprevir
**Drug dependence disorder**<br>Drug dependence was defined as age between 11 and 65 inclusive, and at least one diagnostic code related to drug psychoses, drug dependence, poisoning by analeptics , nondependent abuse of drugs, drug dependence of mothers (antepartum and postpartum condition or complication), or counselling on substance abuse.<br>**Physician Billing Data:** MSP ICD-9 diagnostic codes: starting with 292 304 970 981 3052 3053 3054 3055 3056 3057 3058 3059 6483 7960 9621 9650 9658 9663 9664 9670 9671 9672 9674 9675 9676 9678 9679 9684 9685 9694 9695 9696 9697 9698 9699 9710 9820 9821 9823 9824 9828 9872 9874 9878 9892 V5789 E8500; exact codes 65550 65551 65553 V6542<br>**Hospitalization Data:** DAD1/DAD3/ICD-9-CM diagnostic codes: starting with 292 304 970 981 3052 3053 3054 3055 3056 3057 3058 3059 6483 7960 9621 9650 9658 9663 9664 9670 9671 9672 9674 9675 9676 9678 9679 9684 9685 9694 9695 9696 9697 9698 9699 9710 9820 9821 9823 9824 9828 9872 9874 9878 9892 V5789 E8500 exact codes 65550 65551 65553 V6542<br>DAD2/NACRS/VS/ICD-10-CA diagnostic codes: starting with F11 F12 F13 F14 F15 F16 F18 F19 T40 T52 T53 Z503 Z715; exact codes: O35501 O35503 O35509 P044 R781 R782 R783 R784 R785 T387 T412 T423 T424 T425 T426 T427 T428 T436 T438 T439 T507 T590 T598 X41 X42 X62 Y12 Z040<br>NACRS ICD-10-CA complaint codes: starting with 751, 753
**Elixhauser Comorbidity Index**<br>Using DAD ICD-9 and ICD-10 code data, we calculated a score in which any hospitalization for one of the 31 Elixhauser diagnostic groups was scored as 1.<br>Congestive Heart Failure, Cardiac Arrhythmia, Valvular Disease, Pulmonary Circulation Disorders, Peripheral Vascular Disorders, Hypertension Uncomplicated, Hypertension Complicated, Paralysis, Neurological Disorders, Chronic Pulmonary Disease, Diabetes Uncomplicated, Diabetes Complicated, Hypothyroidism, Kidney Failure, Liver Disease, Peptic Ulcer Disease excluding bleeding, AIDS/HIV, Lymphoma, Metastatic Cancer, Solid Tumor without Metastasis, Rheumatoid Arthritis/collagen, Coagulopathy, Obesity, Weight Loss, Fluid and Electrolyte Disorders, Blood Loss Anemia, Deficiency Anemia, Alcohol Abuse, Drug Abuse, Psychoses, Depression.
**End-stage kidney disease**<br>End-stage kidney disease was defined at the occurrence of nine or more dialysis-related MSP fee item codes, within 90 days, or 1 physician billing code or 1 hospitalization diagnosis or procedure code for kidney transplantation, or 1 urgent care diagnostic code.<br>**Physician Billing Data:** MSP ICD9 fee item codes: 00308, 00323, 00350, 00351, 00352, 00355, 00356, 00358, 00359, 00361, 00390, 33708, 33723, 33750, 33751, 33752, 33755, 33756, 33758, 33759, 33761, 33790, 77400, 10903.<br>**Hospitalization Data:** DAD1/DAD3/ICD9-CM procedure code 675<br>DAD2/NACRS/VS ICD-10-CA diagnostic codes: starting with T861, Z940, Z992, diagnostic code 1PC85
**Hepatitis B virus (HBV) infection**<br>HBV infection was defined at the first occurrence of a case of HBV recorded in iPHIS or PANORAMA; if none recorded, then the first occurrence of 2 MSP (2 physician visits or 2 fee items), 1 hospitalization code or 1 PharmaNet code involving acute or chronic hepatitis B diagnoses with or without hepatic coma and with or without delta agents; inactive carriers; or hepatitis B-specific treatment among those diagnosed at age 18 months or older.<br>**Physician Billing Data:** MSP ICD-9 diagnostic codes: starting with 702, 703, V0261; Fee item codes: 9170, 9171, 9470, 9661, 90675, 90690, 90700, 90831, 90835, 91065, 91210, 91765<br>**Hospitalization Data:** DAD1/DAD3 ICD-9-CM diagnostic codes: starting with 702, 703, V0261; DAD2/NACRS ICD-10-CA diagnostic codes: starting with B16, B180, B181, Z2250.

© 2025 Jeong D et al. *JAMA Network Open*.

**PharmaNet Data:** DIN PIN: 2247823, 02420333, 2282224, 02396955, 02418312, 02430576, 02430584, 02448777, 02453797, 02467232, 02479907, 02485907, 02192691, 2239193, 02239194, 02282216, 02393239, 2288389, 02464241, 2247128, 02403889, 02451980, 02452634, 02453940, 02460173, 02472511, 02479087.
**Hospitalized stroke**<br>Hospitalized stroke, including hospitalized stroke (hemorrhagic, ischemic and transient ischemic attack), was defined at the occurrence of 1 of the following hospitalization diagnostic codes.<br>**Hospitalization Data:** DAD1/ICD-9-CM: starting with 362.3 430, 431, 433.x1, 434.x, 435, or 436. DAD2/ICD-10-CA: starting with G45.0, G45.1, G45.2, G45.3, G45.8, G45.9, H34.0, H34.1, I60, I61, I63 (exclude I63.6), or I64.
**Human immunodeficiency virus (HIV) infection**<br>HIV infection was defined at the first occurrence of 3 MSP or 1 hospitalization for HIV or a positive HIV serologic test, HAISYS or BC Vital Statistics indication (cause of death).<br>**Physician Billing Data:** MSP ICD-9 diagnostic codes: starting with 042, 043, 044, 7953, 7958, 79571, V08.<br>**Hospitalization Data:** DAD1/DAD3 ICD-9-CM diagnostic codes: starting with 042, 043, 044, 7953, 7958, 79571, V08; DAD2/NACRS ICD-10-CA diagnostic codes: starting with B20-B24, B9735, F024, O987, R75, Z21.
**Hypertension**<br>Hypertension was defined as one hospitalization or two physician visits in two years with diagnostic code(s) specified below. The case definition applies to persons aged 20 and older.<br>**Physician Billing Data:** MSP ICD-9 diagnostic codes: starting with 401, 402, 403, 404, 405.<br>**Hospitalization Data:** DAD1/DAD3 ICD-9-CM diagnostic codes: starting with 401, 402, 403, 404, 405; DAD2 ICD-10-CA diagnostic codes: starting with I10, I11, I12, I13, I15.
**Injection drug use**<br>Injection drug use was defined at the occurrence of at least two physician visits, one hospitalization, OR one emergency department visit related to major drug-related diagnoses involving addiction, dependence, and drug-induced mental disorders; illicit drug use most likely to be injecting (e.g. excluding cannabis), or illicit use of prescribed drugs including: hallucinogens, barbiturates/tranquillizers, sedatives, hypnotics, anxiolytics, opioids, cocaine, amphetamine, volatile solvents; or discharge to drug rehabilitation, counselling, and surveillance (11 ≤ diagnosis age ≤ 65).<br>**Physician Billing Data:** MSP ICD-9 diagnostic codes: starting with 292, 970, 3040-42, 3044-49, 3054-57, 3059, 6483, 7960, 9621, 9650, 9658, 9663-64, 9670, 9684-85, 9694, 9696-99, 9700, 9701, 9708, 9709, E8500, or exact codes V6542, 9672, E9397<br>**Hospitalization Data:** DAD1/DAD3 ICD-9-CM: starting with 292, 970, 3040-42, 3044-49, 3054-7, 3059, 6483, 7960, 9621, 9650, 9658, 9663-64, 9670, 9684-85, 9694, 9696-99, 9700, 9701, 9708, 9709, E8500 ,or exact codes V6542, 9672, E9397; NACRS/DAD2 ICD-10-CA: starting with F11, F13-5, F19, Z722 or exact codes R781-82, T387, T400-T406, T408-09, T412, T423-28, T436, T438-39, T507, T4020-23, T4028, T4040-41, T4048; NACRS ICD10 complaint codes: exact codes 751, 753.
**Major adverse cardiac events**<br>Major adverse cardiac events included the following:<br>**Acute myocardial infarction (hospitalized)**, defined at the occurrence of the hospitalization diagnostic codes for Acute Myocardial Infarction:<br>**Hospitalization Data:** DAD1/ICD-9-CM: starting with 410; DAD2/ICD-10-CA: starting with I21.<br>**Angina**, defined as either (i) one hospitalization with an Angina diagnostic code specified below, (ii) one specialist visit with Angina diagnostic code plus one Angina drug prescription (as specified in PharmaNet list below) in one year, or (iii) two physician visits with one Angina prescription in one year.<br>**Hospitalization Data:** DAD1/ICD-9-CM: starting with 413; DAD2/ICD-10-CA: starting with I20.<br>**Physician Billing Data:** MSP ICD-9 diagnostic codes: starting with 413.<br>**PharmaNet Data:** DINPIN numbers: 3662,14214,15954, 15962, 37613, 37621, 104736, 125849, 202959, 202967, 202975, 208973, 243116, 279536, 299790, 342734, 441686, 441694, 442925, 446661, 446688, 446696, 458686, 458694, 476579, 476587, 476595, 476609, 525529, 576174, 584223, 584258, 584266, 590673, 590681, 590738, 590746, 602892, 608785, 614262, 658812, 660655, 670944, 677469, 695726, 725242, 740721, 749362, 749370, 749389, 749397, 754188, 778869, 786640, 786667, 786683, 852384,

© 2025 Jeong D et al. *JAMA Network Open*.

860778, 874213, 874248, 1911902, 1911910, 1911929, 1913921, 1926454, 1926721, 1927809, 1946196, 2011271, 2041715, 2041723, 2042606, 2042614, 2042622, 2044811, 2046156, 2046164, 2058472, 2125218, 2126559, 2145294, 2145308, 2145316, 2162806, 2163527, 2163535, 2164337, 2182734, 2213370, 2230732, 2230733, 2230734, 2231441, 2238998, 2243588, 2272830, 2301288, 2393433, 2407442, 2407450, 2407469, 2407477<br>**Heart failure**, defined as either (i) one hospitalization with diagnostic code for heart failure specified below, or (ii) two physician visits with diagnostic code specified below in one year.<br>**Hospitalization Data:** DAD1/ICD-9-CM: starting with 428; DAD2/ICD-10-CA: starting with I50.<br>**Physician Billing Data:** MSP ICD-9 diagnostic codes: starting with 428.<br>**Peripheral vascular disease**, defined as either (i) one hospitalization with diagnostic codes used to identify peripheral arterial disease specified below, or (ii) two physician visits with diagnostic codes specified below.<br>**Hospitalization Data:** DAD1/ICD-9-CM: 440.24, 440.21, 440.23, 440.20, 440.22, 443.9, 440.9; DAD2/ICD-10-CA: I70.2, I73.9, I70.9. Procedural codes 1WK93 (Canadian Classification of Health Interventions) excluding records with ICD-10 diagnosis codes: C40, D16, D48.0, D48.2, Q65-79, S70-S99, T20-T32; 3KG10 + BL, 3KG10 + LL, 3KG20, 3KG30, 3KG40.<br>**Physician Billing Data**: MSP ICD-9 diagnostic codes: 440.24, 440.21, 440.23, 440.20, 440.22, 443.9, 440.9.<br>**Percutaneous transluminal coronary angioplasty**, defined as one hospitalization with procedure codes for percutaneous transluminal coronary angioplasty specified below.<br>**Hospitalization data:** Procedural codes: ICD9 starting with 4802, 4803; ICD10 starting with 1IJ50, 1IJ57G.<br>**Coronary artery bypass graft**, defined as one hospitalization with procedure codes for coronary artery bypass graft specified below.<br>**Hospitalization data:** Procedural codes: ICD9 starting with 4811, 4812, 4813, 4814, 4815, 4816, 4817, 4819; ICD10 starting with 1IJ57LA, 1IJ57VS, 1IJ76.<br>**Stroke**, including hospitalized stroke (hemorrhagic, ischemic and transient ischemic attack), defined at the occurrence of 1 of the following hospitalization diagnostic codes.<br>**Hospitalization Data:** DAD1/ICD-9-CM: starting with 362.3 430, 431, 433.x1, 434.x, 435, or 436. DAD2/ICD-10-CA: starting with G45.0, G45.1, G45.2, G45.3, G45.8, G45.9, H34.0, H34.1, I60, I61, I63 (exclude I63.6), or I64.
**Major mental illness**<br>Major mental illness was defined at the first occurrence of one hospitalization diagnostic code OR two physician diagnostic codes from a psychiatrist visit for schizophrenic, bipolar, delusional, nonorganic psychotic, adjustment, anxiety, dissociative, personality and major depressive disorders.<br>**Physician Billing Data:** MSP ICD-9 diagnostic codes: starting with 295-298, 300-301, 308-309, 311 or exact code 50B AND claim specialty = 3<br>**Hospitalization Data:** DAD1/DAD3 ICD-9-CM diagnostic codes: starting with 295-298, 300-301, 308-309, 311; DAD2/NACRS/VS ICD-10-CA diagnostic codes: starting with F20-F25, F28-F34, F38-F45, F48, F60-F61, F99, Y495
**Material and Social Deprivation Quintiles**<br>The Québec Index of Material and Social Deprivation (1) was calculated based on individuals' 6-digit postal code. The deprivation index combines six indicators related to health and welfare that represent material or social deprivation and are available by enumeration area in Canadian census data: 1) proportion of persons without high-school diploma 2) ratio of employment to population 3) average income 4) proportion of persons separated, divorced, widowed 5) the proportion of single-parent families 6) proportion of people living alone.
**Neurocognitive disorders**<br>Neurocognitive disorders were defined as at least 1 physician billing or hospitalization code related to dementia, cerebral degenerations, delirium, Alzheimer's disease.<br>**Physician Billing Data:** MSP ICD-9 diagnostic codes: starting with 290, 294, 331 or 33182.<br>**Hospitalization Data:** DAD1/DAD3/ICD-9-CM diagnostic codes: starting with 290, 294,331 or 33182. DAD2/NACRS/VS ICD-10-CA diagnostic codes: starting with F01, F03, F04, F05, F06, F09, G30, G3183
**Non-alcoholic fatty liver disease**

© 2025 Jeong D et al. *JAMA Network Open*.

Non-alcoholic fatty liver disease was defined by at least one physician billing or hospitalization code related to other chronic non-alcoholic liver disease, other specified inflammatory liver diseases, or fatty (change of) liver, not elsewhere classified.<br>**Physician Billing Data:** MSP ICD-9 diagnostic codes: exact 5718<br>**Hospitalization Data:** NACRS/DAD1/DAD3/ICD-9-CM diagnostic codes: exact 5718<br>DAD2/NACRS/VS/ICD-10-CA diagnostic codes: exact K758, K760
**Obesity**<br>Obesity was defined as at least two physician visit codes, one hospitalization code, or one NACRS code, involving obesity, obesity complicating pregnancy, screening for obesity, obese BMI, bariatric surgery, gastrectomy, or gastric partitioning surgery; or one PharmaNet code involving obesity-related prescriptions for a minimum duration of 30 days.<br>**Physician codes** MSP/ICD-9 diagnostic codes starting with 2780 6491 6492 V853 V854; or exact codes V4586 V778; Fee items: exact codes 1169, 7003<br>**Hospitalization codes** DAD1/DAD3/ICD-9 diagnostic codes starting with 2780 6491 6492 V853 V854; or exact codes V4586 V778 or exact procedure codes 558 5693 7652<br>DAD2/ICD-10 exact procedure codes: 1ZZ35CAA8 1ZZ35HAA8 1ZZ35YAA8<br>DAD2/NACRS/ICD-10 diagnostic codes starting with E66 Z683 Z684<br>**PharmaNet codes**: DIN PIN: 18058 26506 133957 227927 227935 285544 344397 411426 418382 432407 449628 779490 891762 891770 1914057 1916831 2043351 2047675 2052490 2235390 2235395 2240325 2243163 2243164 2318970 2318989 2337614 2337622 2472945
**Opioid Agonist Therapy**<br>Opioid agonist therapy was defined as at least one MSP fee item code involving methadone, buprenorphine, or naloxone treatment or one DIN PIN in PharmaNet data involving OAT treatments<br>**Physician Billing Data:** MSP ICD-9 diagnostic codes: exact code 39<br>**PharmaNet Data:** DIN PIN numbers: 999776 999792 999793 2241377 2242963 2242964 2295695 2295709 2408090 2408104 2424851 2424878 2453908 2453916 2468085 2468093 2474921 2481979 2483092 2495872 2495880 22123346 22123347 22123348 22123349 22123357 66999990 66999991 66999992 66999993 66999997 66999998 66999999 67000000 67000001 67000002 67000003 67000004 67000005 67000006 67000007 67000008 67000009 67000010 67000011 67000012 67000013 67000014 67000015 67000016 67000017 67000018 67000019 67000020 2495783 9858127 9858128, 2502313, 2502348, 2502321, 2502356, 2483084
**Statin use**<br>Statin use was defined as the first occurrence for any statin dispensation using the following DIN PIN in PharmaNet.<br>**PharmaNet Data:** DIN PIN: 2355639, 2295261, 2391082, 2433796, 2351765, 2346494, 2396432, 2288362, 2397862, 2399385, 2230711, 2313472, 2313448, 2373238, 2302683, 2355620, 2391058, 2310902, 2295288, 2295318, 2387921, 2397897, 2392933, 2313456, 2435306, 2435314, 2454033, 2302691, 2324946, 2398141, 2364441, 2373211, 2433761, 2346516, 2422794, 2350327, 2399393, 2376326, 2384817, 2333724, 2302713, 2351773, 2407256, 2417960, 2348624, 2348721, 2324962, 2398117, 2399377, 2376288, 2389541, 2399504, 2346508, 2422778, 2348705, 2324954, 2364468, 2392976, 2230714, 2373246, 2433788, 2396440, 2422786, 2417952, 2310899, 2310910, 2411385, 2387905, 2392941, 2243097, 2384795, 2384809, 2389576, 2333759, 2399482, 2302675, 2407272, 2417936, 2288370, 2411369, 2387891, 2364476, 2364484, 2333740, 2399490, 2355612, 2346486, 2391074, 2348640, 2411377, 2398125, 2454017, 2313758, 2313723, 2350335, 2288354, 2387913, 2384787, 2351757, 2417944, 2310929, 2348659, 2350319, 2399407, 2433818, 2389568, 2396459, 2348632, 2350297, 2435322, 2333732, 2454025, 2313715, 2396424, 2407280, 2422751, 2390182, 2313707, 2348748, 2398133, 2373203, 2355647, 2407264, 2288346, 2295296, 2397870, 2313464, 2435292, 2454041, 2351781, 2391066, 2324970, 2348713, 2411350, 2397889, 2392968, 2230713, 2376318, 2376296, 2389584, 2362767, 2411261, 2427702, 2362783, 2411253, 2273314, 2362805, 2362813, 2400774, 2362821, 2273241, 2362775, 2273268, 2273306, 2400758, 2362791, 2411334, 2273284, 2400782, 2400790, 2427737, 2427710, 2400820, 2404249, 2404257, 2362759, 2411288, 2273233, 2411296, 2411318, 2411342, 2273276, 2400812, 2404230, 2411326, 2273292, 2404222, 2427729, 2400804, 2400839, 2243223, 2237326, 2237325, 2241466, 2400235, 2061562, 2061570, 2299224, 2250527, 2299232, 2400243, 2247232, 2364247, 2246014, 2239956, 2252929, 2272288, 2353229, 2220172, 795852, 2331845, 2243127, 2248572, 2246542, 2246989, 2231434,

© 2025 Jeong D et al. *JAMA Network Open*.

2247056, 2344335, 2243129, 2245822, 2341476, 2312689, 2246543, 2353237, 2246013, 2312670, 2248573, 2220180, 2267969, 2272296, 2247231, 2246990, 2364239, 2344343, 2245823, 2247537, 2252937, 2331837, 795860, 2267977, 2247536, 2239955, 2341484, 2247057, 2293501, 2270439, 2270447, 2270429, 2242866, 2243825, 2247858, 2430983, 2389738, 2248184, 2243506, 2317486, 2340259, 2243824, 2427257, 2440644, 2432048, 2257092, 2249731, 2265613, 2270234, 2301806, 2379791, 2310775, 2274523, 2274507, 2445379, 893757, 2257106, 2247009, 2370875, 2246932, 2340267, 2379783, 2379805, 2345757, 2345730, 2458977, 2340275, 2331586, 2364417, 2249782, 2345749, 2330962, 2243508, 2389746, 893749, 2257114, 2249758, 2237374, 2265621, 2301792, 2430975, 2432064, 2342340, 2244351, 2270242, 2364433, 2332191, 2247656, 2247010, 2256851, 2246930, 2247856, 2440652, 2249723, 2440660, 2247657, 2284448, 2222051, 2243826, 2427265, 2446286, 2243507, 2244350, 2342332, 2330970, 2301814, 2270250, 2237375, 2331594, 2356554, 2284421, 2389703, 2317451, 2242865, 2249774, 2242867, 2247857, 2274515, 2445395, 2330954, 2458985, 2256878, 2256886, 2364425, 2246931, 2310759, 2332213, 2445409, 2247008, 2247655, 2248183, 2248182, 2458993, 2370891, 2237373, 2331608, 2310767, 2332205, 2432056, 2342324, 2446251, 2356546, 2244352, 2370905, 2249766, 2265648, 2430967, 2446278, 2356562, 2284456, 2317478, 2272423, 2272431, 2272415, 2413051, 2413086, 2380064, 2338734, 2339773, 2381192, 2386739, 2444968, 2397803, 2378531, 2378566, 2380056, 2247163, 2389436, 2445441, 2411644, 2389053, 2381281, 2339781, 2337975, 2399199, 2247162, 2385538, 2385546, 2389355, 2397838, 2405628, 2382644, 2339765, 2354616, 2337991, 2438917, 2445425, 2391279, 2397781, 2354608, 2338009, 2389061, 2385511, 2389401, 2438925, 2438933, 2433141, 2381303, 2433125, 2410966, 2445417, 2391252, 2413078, 2405636, 2405644, 2442590, 2442604, 2265540, 2410958, 2390558, 2380013, 2339803, 2399180, 2442582, 2442574, 2444976, 2343533, 2381206, 2410974, 2411628, 2411652, 2378558, 2338750, 2381273, 2381184, 2385554, 2444984, 2391260, 2386712, 2382660, 2354624, 2378523, 2389428, 2343517, 2390523, 2411636, 2386720, 2382679, 2381176, 2399164, 2389037, 2247164, 2438941, 2390507, 2405652, 2399172, 2389045, 2380102, 2433133, 2433168, 2444992, 2445433, 2343525, 2390531, 2391287, 2386704, 2382652, 2338726, 2338742, 2354632, 2337983, 2410931, 2343509, 2397811, 2413108, 2381265, 2247531, 2252643, 2253747, 2338866, 2247076, 2341956, 2349949, 2349965, 2364034, 2378884, 2372967, 2250152, 2375648, 2386321, 2269287, 2248107, 884324, 2253712, 2247075, 2397668, 2397633, 2265672, 2342456, 2375044, 2376059, 2375060, 2284774, 884359, 2253739, 2281619, 2265915, 2281643, 2181562, 2341980, 2377640, 2329182, 2329174, 2331985, 2375613, 2376075, 2247068, 2331039, 2343150, 2300915, 2300907, 2247077, 2400278, 2377675, 2377284, 2246584, 2248103, 2372975, 2246737, 2248106, 2338904, 2400251, 2397684, 2374676, 2265680, 2372959, 2247299, 2376083, 2376067, 2246583, 884332, 2247012, 2253755, 2265885, 2300923, 2400286, 2400308, 2397641, 2329158, 2329166, 2246585, 2375591, 2376040, 2247070, 2386313, 2246582, 2250144, 2284758, 2240332, 2331020, 2281627, 2281635, 2364018, 2338882, 2378922, 2265664, 2379538, 2379554, 2329131, 2247069, 2405164, 2247300, 2253720, 2281570, 2265907, 2247831, 2343142, 2300974, 2247533, 2247224, 2374633, 2374625, 2342480, 2331993, 2332000, 2375052, 2386305, 2405148, 2405156, 2265893, 2247830, 2253771, 2341964, 2378892, 2253798, 2374641, 2377314, 2331969, 2247297, 2405180, 2247301, 2247222, 2247534, 2343169, 2300931, 2265877, 2341999, 2349922, 2364050, 2378906, 2377659, 2379503, 2247011, 2375605, 2375036, 2248104, 2247298, 2375621, 2284731, 2284766, 2250187, 2247220, 2252651, 2281651, 2338890, 2338874, 2400294, 2247223, 2374668, 2342464, 2247067, 2269279, 2247071, 2269252, 2247828, 2247833, 2253704, 2247221, 2253690, 2252635, 2341972, 2349930, 2281546, 2281562, 2342499, 2372940, 2332019, 2250179, 2331063, 2253763, 2364026, 2364042, 2265656, 2265699, 2375079, 2269260, 2281554, 2379511, 2331055, 2343185, 2247072, 2247535, 2252619, 2349957, 2377306, 2377292, 2342472, 2379546, 2372932, 884340, 2386291, 2405172, 2248105, 2247078, 2331047, 2281589, 2247827, 2378914, 2269295, 2386348, 2247014, 2252678, 2343177, 2377632, 2377667, 2247532, 2397676, 2377322, 2284723, 2250160, 2247015, 2247013.
**Type 2 diabetes mellitus (T2DM)**<br>Diabetes mellitus was defined as two physician billing codes within 2 years, one hospitalization code, one emergency department visit, or one vital statistics code related to type 2 diabetes mellitus (excluding gestational diabetes and type 1 diabetes), OR one physician fee item code related to chronic kidney disease, operative tissue ablation and reconstruction of atria, or a health and behavior assessment OR two or more pharmacy dispensations within 1 year.<br>**Physician Billing Data:** MSP ICD-9 diagnostic codes: starting with 249, 250; Fee items: exact codes 585, 14050, 14250, 33255, 33256, 96150.

© 2025 Jeong D et al. *JAMA Network Open*.

Hospitalization Data: DAD1/DAD3 ICD-9-CM diagnostic codes: starting with 249, 250; DAD2/NACRS/VS ICD-10-CA diagnostic codes: starting with E10, E11, E12, E13, E14; procedure codes: DAD2 exact codes 1ZZ35CAB1, 1ZZ35HAB1, 1ZZ35YAB1

PharmaNet Data: DIN PIN: 5894 6009 12556 12564 12599 12602 12610 13730 13889 15598 21350 21849 24708 24716 93033 156663 156728 178543 209872 209937 244449 274119 274127 275409 275417 275425 312711 312762 314552 377937 399302 420336 430986 431168 446564 446572 446580 446599 446602 446610 454753 480290 480304 513644 514535 514551 539201 539244 542911 542938 542946 546348 552259 552267 552275 554820 586714 586773 587737 612162 612170 612189 612197 612200 612219 612227 612235 612243 612251 612278 612359 614416 628301 632651 632678 632686 632694 644358 646148 648094 650935 720933 720941 723789 733075 765996 773654 795879 808733 808741 889091 889105 889113 889121 999717 1900927 1900935 1913654 1913662 1913670 1913689 1934066 1934074 1934082 1934090 1934104 1934112 1959212 1959220 1959239 1959352 1959360 1962639 1962647 1962655 1962663 1985930 1985949 1985957 1985965 1985973 1985981 1986085 1986791 1986805 1986813 1986821 1987534 1987542 1987828 1987836 2020734 2020742 2022230 2022249 2024217 2024225 2024233 2024241 2024268 2024276 2024284 2024292 2024306 2024314 2024322 2024403 2024446 2025248 2025256 2045710 2084341 2085887 2099233 2147521 2147548 2148765 2155850 2162822 2162849 2167786 2188902 2190885 2190893 2220628 2223562 2224550 2224569 2224771 2224798 2226804 2226812 2228920 2228939 2229516 2229517 2229519 2229595 2229596 2229656 2229704 2229705 2229785 2229994 2230026 2230027 2230036 2230037 2230443 2230444 2230475 2230670 2230671 2231058 2231095 2231096 2231389 2233562 2233999 2234513 2234514 2236543 2236548 2236733 2236734 2236985 2236986 2237531 2238103 2238469 2238470 2238471 2238698 2238827 2239081 2239214 2239474 2239475 2239476 2239924 2239925 2239926 2240294 2240295 2240297 2241111 2241112 2241113 2241114 2241283 2241310 2242095 2242096 2242572 2242573 2242574 2242589 2242726 2242783 2242793 2242794 2242931 2242974 2242987 2244353 2245247 2245272 2245273 2245274 2245397 2245438 2245439 2245440 2245689 2246820 2246821 2246964 2246965 2247085 2247086 2247087 2248008 2248009 2248210 2248440 2248441 2248453 2251930 2252945 2252953 2254719 2257726 2257734 2258781 2258803 2258811 2265435 2265443 2265575 2265583 2268493 2268507 2269031 2269058 2269589 2269597 2269600 2269619 2271842 2273101 2273128 2273136 2273756 2273764 2273772 2274248 2274256 2274264 2274272 2274914 2274922 2274930 2275864 2275872 2276410 2279061 2279088 2279126 2279460 2279479 2279487 2284545 2284553 2284782 2284790 2287072 2294338 2294346 2294400 2295377 2295385 2295393 2297795 2297906 2297914 2297922 2298279 2298287 2298295 2300451 2301423 2301431 2301458 2302861 2302888 2302896 2302942 2302950 2302977 2303124 2303132 2303140 2303442 2303450 2303469 2303922 2305062 2306166 2306174 2306182 2307170 2307189 2307197 2307553 2307561 2307588 2307634 2307642 2307650 2307669 2307677 2307723 2312050 2312069 2312077 2313596 2314894 2314908 2316544 2320754 2320762 2320770 2321475 2321483 2321491 2326329 2326337 2326345 2326477 2326485 2326493 2331519 2331527 2333554 2333856 2333864 2333872 2334437 2334445 2336316 2339110 2339129 2339587 2339595 2340763 2340771 2341522 2341603 2343606 2343614 2345366 2345374 2345382 2345854 2345862 2348578 2350459 2350467 2351056 2351064 2353377 2353385 2354144 2354152 2354160 2354349 2354357 2354365 2354926 2354934 2354942 2355663 2355671 2355698 2356422 2357453 2357461 2357488 2357887 2357895 2357909 2357917 2357925 2361264 2361272 2361809 2361817 2363232 2363240 2363259 2363518 2363704 2363712 2364506 2364514 2365286 2365294 2365529 2365537 2366347 2366355 2366363 2370921 2373270 2373289 2373297 2374013 2374021 2374048 2374587 2374595 2375842 2375850 2375869 2375877 2377209 2378043 2378051 2378116 2378124 2378620 2378639 2378841 2378868 2379767 2379775 2380196 2380218 2380722 2380730 2384906 2384914 2384922 2385341 2385368 2388766 2388774 2388839 2388847 2389169 2389177 2389185 2389290 2389304 2389312 2391600 2397307 2403250 2403269 2403277 2403366 2403374 2403382 2403412 2403420 2403439 2403447 2405067 2406020 2406039 2407124 2408228 2408236 2409283 2409291 2412829 2415089 2415968 2415976 2415984 2416786 2416794 2416808 2417049 2417057 2417065 2417189 2417197 2417200 2417219 2417227 2417235 2418002 2418010 2418029 2419300 2419319 2419327 2419335 2419343 2419351 2421674 2421682 2421690 2421828 2421836 2423286 2423294 2424258 2424266 2424274 2425483 2425491 2429764 2429772 2434121 2434148 2434156 2435462 2435470 2437899 2438275 2438283 2438658 2439328

© 2025 Jeong D et al. *JAMA Network Open*.

2439611 2441829 2443635 2443643 2443937 2443945 2444844 2444852 2444933 2444941 2446065 2448599 2448602 2448610 2449390 2449404 2449765 2449935 2449943 2455404 2455412 2455420 2455439 2455447 2455455 2456575 2456583 2456591 2456605 2456613 2456621 2459183 2459752 2459760 2460408 2460416 2460424 2460653 2461323 2461331 2461528 2463571 2464276 2464284 2464349 2466864 2467879 2467887 2469871 2469898 2469901 2470152 2471469 2471477 2474875 2475510 2475529 2475901 2475928 2476215 2476223 2476231 2476258 2477394 2477408 2477416 2477424 2478293 22303140 44123021 44123024 44123025 44123026 44123028 44123029 44123033 44123034 44123035 44123036 44123037 44123038 44123040 44123042 44123043 44123044 44123045 44123046 44123047 44123048 44123049 44123051 44123052 44123053 44123055 44123056 44123057 44123058 44123059 44123060 44123061 44123062 44123063 44123064 44123065 45230001 45230002 45230003 45230004 45230005 45230006 45230007 45230008 45230009 45230010 45230011 45230012 45230013 45230014 47450001 47450002 47450003 47450004 47450005 47450006 47450007 48123021 48123024 48123025 48123026 48123028 48123029 48123033 48123034 48123035 48123036 48123037 48123038 48123040 48123042 48123043 48123044 48123045 48123046 48123047 48123048 48123049 48123051 48123052 48123053 48123055 48123056 48123057 48123058 48123059 48123060 48123061 48123062 48123063 48123064 48123065 66123203 66124134 66124135 66124215 66124225 66124232 66124582 66127961 2493799 2493780 2456966 2456974 2463601 2463628 2468603 2468611 2479575 2479583 2506564 2506572 2507471 2507498 45230017 45230018 45230019 46340038 46340039 46340040 2423294 2456966 2456974 2459183 2459752 2459760 2460408 2460416 2460424 2460653 2461323 2461331 2461528 2463571 2463601 2463628 2464276 2464284 2464349 2466864 2467879 2467887 2468603 2468611 2469871 2469898 2469901 2470152 2471469 2471477 2474875 2475510 2475529 2475901 2475928 2476215 2476223 2476231 2476258 2477394 2477408 2477416 2477424 2478293 2479575 2479583 2485664 2493373 2493780 2493799 2494078 2494086 2494418 2494442 2497581 2497603 2497611 2506564 2506572 2507471 2507498

Abbreviations: DAD, discharge abstracts database; DIN, drug identification number; ICD, international classification of diseases; MSP, medical services plan; PIN, product information number.

© 2025 Jeong D et al. *JAMA Network Open*.

eTable 3. Prevalence of extrahepatic manifestations at baseline among study participants by treatment status, overall, stratified by age and genotype

	Untreated	Treated	p-value[a]	SVR	No-SVR	p-value[b]
**Overall study population**	*n=10,237*	*n=12,339*		*n=11,953*	*n=386*	
CKD & ESKD	1,111 (10.9%)	1,272 (10.3%)	0.1854	1,237 (10.4%)	35 (9.1%)	0.4168
T2DM	1,311 (12.8%)	2,103 (17.0%)	<.0001	2,037 (17.0%)	66 (17.1%)	0.9802
Stroke	380 (3.7%)	330 (2.7%)	<.0001	320 (2.7%)	10 (2.6%)	0.9183
MACE	1,290 (12.6%)	1,434 (11.6%)	0.0228	1,385 (11.6%)	49 (12.7%)	0.5077
NCD	1,092 (10.7%)	949 (7.7%)	<.0001	913 (7.6%)	36 (9.3%)	0.2376
**Born on or before 1959**	*n=4,283*	*n=6,603*		*n=6,418*	*n=185*	
CKD &	654 (15.3%)	716 (10.8%)	<.0001	697 (10.9%)	19 (10.3%)	0.8093
ESKD T2DM	931 (21.7%)	1,413 (21.4%)	0.6852	1,363 (21.2%)	50 (27.0%)	0.0604
Stroke	278 (6.5%)	222 (3.4%)	<.0001	216 (3.4%)	6 (3.2%)	0.9255
MACE	979 (22.9%)	1,047 (15.9%)	<.0001	1,015 (15.8%)	32 (17.3%)	0.5946
NCD	709 (16.6%)	520 (7.9%)	<.0001	503 (7.8%)	17 (9.2%)	0.4934
**Born after 1959**	*n=5,954*	*n=5,736*		*n=5,535*	*n=201*	
CKD &	457 (7.7%)	556 (9.7%)	0.0001	540 (9.8%)	16 (8.0%)	0.3973
ESKD T2DM	380 (6.4%)	690 (12.0%)	<.0001	674 (12.2%)	16 (8.0%)	0.0730
Stroke	102 (1.7%)	~109 (1.9%)	0.4646	104 (1.9%)	≤5	0.6285
MACE	311 (5.2%)	387 (6.7%)	0.0006	370 (6.7%)	17 (8.5%)	0.3365
NCD	383 (6.4%)	429 (7.5%)	0.0225	410 (7.4%)	19 (9.5%)	0.2880
**HCV GT1**	*n=4,528*	*n=8,058*		*n=7,837*	*n=221*	
CKD &	545 (12.0%)	810 (10.1%)	0.0005	794 (10.1%)	16 (7.2%)	0.1536
ESKD T2DM	599 (13.2%)	1,397 (17.3%)	<.0001	1353 (17.3%)	44 (19.9%)	0.3069
Stroke	180 (4.0%)	~204 (2.5%)	<.0001	199 (2.5%)	≤5	0.8170
MACE	626 (13.8%)	954 (11.8%)	0.0012	927 (11.8%)	27 (12.2%)	0.8646
NCD	520 (11.5%)	599 (7.4%)	<.0001	582 (7.4%)	17 (7.7%)	0.8845
**HCV GT3**	*n=1,942*	*n=2,592*		*n=2,462*	*n=130*	
CKD &	207 (10.7%)	289 (11.1%)	0.6246	274 (11.1%)	15 (11.5%)	0.8864
ESKD T2DM	199 (10.3%)	391 (15.1%)	<.0001	373 (15.2%)	18 (13.9%)	0.6937
Stroke	52 (2.7%)	~73 (2.8%)	0.7795	68 (2.8%)	≤5	0.4563

© 2025 Jeong D et al. *JAMA Network Open*.

MACE	168 (8.7%)	236 (9.1%)	0.6150	220 (8.9%)	16 (12.3%)	0.2025
NCD	188 (9.7%)	236 (9.1%)	0.5229	220 (8.9%)	16 (12.3%)	0.2025

Abbreviations: CKD, chronic kidney disease; DAA, direct-acting antivirals; ESKD, end-stage kidney disease; GT, genotype; MACE, major adverse cardiac events; NCD, neurocognitive disorders; SVR, sustained virologic response; T2DM, type 2 diabetes mellitus

[a]Mantel-Haenszel test (categorical variables) was used for comparison
[b]Fisher's exact test was used for comparison

© 2025 Jeong D et al. *JAMA Network Open*.

eTable 4. Comparison of baseline characteristics between 'Untreated', 'Treated & SVR' and 'Treated & No-SVR' groups in the study population and in the inverse probability of treatment weights (IPTW)-weighted dataset

	Unweighted				Weighted with IPTW			
	Untreated (n=10237)	SVR (n=11953)	No-SVR (n=386)	SMD	Untreated (n=9870.9)	SVR (n=11597.2)	No-SVR (n=268.3)	SMD
**Sex (%)**				0.111				0.052
Male	6884 (67.2)	7785 (65.1)	281 (72.8)		6520.4 (66.1)	7746.1 (66.8)	186.9 (69.7)	
**Birth year**				0.142				0.028
Mean (SD)	1963 (13)	1960 (11)	1963 (13)		1962 (12)	1962 (12)	1961 (10)	
**Age at first HCV diagnosis**				0.126				0.014
Mean (SD)	41.0 (12.6)	42.9 (11.3)	40.6 (12.7)		41.9 (11.9)	41.9 (11.7)	41.7 (10.4)	
**Annual average healthcare visits pre-treatment[a]**				0.341				0.041
0 to 6	3369 (32.9)	2339 (19.6)	59 (15.3)		2588.0 (26.2)	2919.6 (25.2)	69.6 (25.9)	
7 to 13	2094 (20.5)	3394 (28.4)	82 (21.2)		2453.4 (24.9)	2878.1 (24.8)	68.5 (25.5)	
14 to 27	2223 (21.7)	3341 (28.0)	117 (30.3)		2384.3 (24.2)	2909.7 (25.1)	68.6 (25.6)	
28+	2551 (24.9)	2878 (24.1)	128 (33.2)		2445.3 (24.8)	2889.1 (24.9)	61.6 (22.9)	
**Annual average healthcare visits post-treatment[b]**				0.471				0.038
0 to 2	3804 (37.2)	1662 (13.9)	74 (19.2)		2471.6 (25.0)	2774.0 (23.9)	67.2 (25.1)	
3 to 11	1961 (19.2)	3807 (31.8)	77 (19.9)		2532.2 (25.7)	3048.5 (26.3)	73.0 (27.2)	
12 to 31	1819 (17.8)	3677 (30.8)	97 (25.1)		2387.3 (24.2)	2907.5 (25.1)	64.2 (23.9)	
32+	2649 (25.9)	2807 (23.5)	138 (35.8)		2476.7 (25.1)	2867.1 (24.7)	63.9 (23.8)	
**Ethnicity (%)**				0.066				0.073
Other[c]	9694 (94.7)	11201 (93.7)	370 (95.9)		9318.7 (94.4)	10939.9 (94.3)	258.7 (96.4)	
East Asian	249 (2.4)	367 (3.1)	8 (2.1)		258.8 (2.6)	317.5 (2.7)	5.5 (2.1)	
South Asian	294 (2.9)	385 (3.2)	8 (2.1)		293.4 (3.0)	339.8 (2.9)	≤5	
**Material deprivation (%)**				0.155				0.076
Q1 (most privileged)	1321 (12.9)	1754 (14.7)	49 (12.7)		1345.0 (13.6)	1628.7 (14.0)	32.0 (11.9)	
Q2	1241 (12.1)	1841 (15.4)	54 (14.0)		1359.3 (13.8)	1637.0 (14.1)	36.6 (13.6)	
Q3	1400 (13.7)	2082 (17.4)	62 (16.1)		1508.5 (15.3)	1836.2 (15.8)	42.6 (15.9)	
Q4	2115 (20.7)	2615 (21.9)	88 (22.8)		2099.0 (21.3)	2462.3 (21.2)	65.5 (24.4)	
Q5 (most deprived)	4091 (40.0)	3567 (29.8)	130 (33.7)		3476.4 (35.2)	3941.6 (34.0)	88.4 (33.0)	
Unknown	69 (0.7)	94 (0.8)	≤5		82.8 (0.8)	91.4 (0.8)	≤5	

© 2025 Jeong D et al. *JAMA Network Open*.

**Social deprivation (%)**				0.171				0.071
Q1 (most privileged)	754 (7.4)	1065 (8.9)	22 (5.7)		771.3 (7.8)	956.8 (8.3)	16.4 (6.1)	
Q2	1046 (10.2)	1502 (12.6)	44 (11.4)		1092.4 (11.1)	1337.2 (11.5)	33.7 (12.5)	
Q3	1270 (12.4)	1910 (16.0)	63 (16.3)		1365.7 (13.8)	1669.7 (14.4)	38.7 (14.4)	
Q4	1698 (16.6)	2194 (18.4)	58 (15.0)		1761.8 (17.8)	2014.6 (17.4)	46.2 (17.2)	
Q5 (most deprived)	5400 (52.7)	5188 (43.4)	196 (50.8)		4797.0 (48.6)	5527.4 (47.7)	130.1 (48.5)	
Unknown	69 (0.7)	94 (0.8)	≤5		82.8 (0.8)	91.4 (0.8)	≤5	
**HCV genotype (%)**				0.291				0.033
Genotype 1	4528 (44.2)	7837 (65.6)	221 (57.3)		5445.0 (55.2)	154.6 (57.6)	6585.2 (56.8)	
Other/unknown[d]	5709 (55.8)	4116 (34.4)	165 (42.7)		4425.9 (44.8)	5012.0 (43.2)	113.7 (42.4)	
**HIV infection (%)**	501 (4.9)	957 (8.0)	48 (12.4)	0.181	624.2 (6.3)	792.7 (6.8)	15.2 (5.7)	0.032
**HBV infection (%)**	588 (5.7)	932 (7.8)	32 (8.3)	0.067	653.9 (6.6)	820.3 (7.1)	15.8 (5.9)	0.032
**Hypertension (%)**	2556 (25.0)	3942 (33.0)	118 (30.6)	0.118	2868.0 (29.1)	3399.7 (29.3)	79.9 (29.8)	0.011
**Statin use (%)**	1170 (11.4)	1636 (13.7)	42 (10.9)	0.057	1297.0 (13.1)	1436.6 (12.4)	28.9 (10.8)	0.049
**Obesity (%)**	270 (2.6)	429 (3.6)	15 (3.9)	0.047	303.9 (3.1)	368.1 (3.2)	6.3 (2.3)	0.034
**NAFLD (%)**	43 (0.4)	129 (1.1)	≤5	0.051	54.0 (0.5)	92.1 (0.8)	≤5	0.061
**Cirrhosis (%)**	847 (8.3)	1871 (15.7)	92 (23.8)	0.29	1090.8 (11.1)	1469.8 (12.7)	32.8 (12.2)	0.033
**Major mental illness (%)**	4399 (43.0)	4488 (37.5)	186 (48.2)	0.144	4013.3 (40.7)	4656.6 (40.2)	116.4 (43.4)	0.044
**Alcohol use disorder (%)**	4086 (39.9)	3868 (32.4)	156 (40.4)	0.112	3644.8 (36.9)	4223.2 (36.4)	96.6 (36.0)	0.013
**Drug dependence disorder (%)**	6632 (64.8)	5803 (48.5)	240 (62.2)	0.221	5632.5 (57.1)	6480.4 (55.9)	148.7 (55.4)	0.022
**Injection drug use (%)**	5435 (53.1)	4572 (38.2)	204 (52.8)	0.201	4556.7 (46.2)	5224.3 (45.0)	125.2 (46.7)	0.022
**Opioid agonist therapy (%)**	3930 (38.4)	3308 (27.7)	160 (41.5)	0.195	3293.0 (33.4)	3778.8 (32.6)	82.3 (30.7)	0.038
**Elixhauser Comorbidity Index (%)**				0.238				0.02
0	3187 (31.1)	4043 (33.8)	76 (19.7)		3245.3 (32.9)	3807.0 (32.8)	85.5 (31.9)	
1	1851 (18.1)	2443 (20.4)	77 (19.9)		1830.6 (18.5)	2204.7 (19.0)	50.2 (18.7)	
≥2	5199 (50.8)	5467 (45.7)	233 (60.4)		4794.9 (48.6)	5585.5 (48.2)	132.7 (49.5)	

Stabilized inverse probability of treatment weights (IPTW) were estimated for the average treatment effect (ATE). PS model included sex, birth year (continuous), age at first HCV diagnosis (continuous), annual average healthcare visits from HCV diagnosis to DAA treatment (categorized as 0 to 6, 7 to 13, 14 to 27, 28+), annual average healthcare visits from 6 months post-treatment to end of study (categorized as 0 to 2, 3 to 11, 12 to 31, 32+), ethnicity, material deprivation quintiles, social deprivation quintiles, HCV genotype (categorized as genotype 1 or other), baseline diagnosis of: HBV and HIV infection, hypertension, statin use, obesity, NAFLD, cirrhosis, major mental illness, alcohol use disorder, drug dependence disorder, injection drug use, opioid agonist therapy, and Elixhauser Comorbidity Index (in categories of 0, 1 or 2 or more).

© 2025 Jeong D et al. *JAMA Network Open*.

[a]Healthcare visits were assessed with number of times any physician billing code from Medical Services Plan or hospitalization code from Discharge Abstract Database was recorded for each individual; from the date of first HCV diagnosis to last DAA treatment initiation (or the same date for matched untreated individual). Categories were created using 25th, 50th and 75th percentiles based on average number of healthcare visits coded for the overall study population
[b]Healthcare visits were assessed with number of times any physician billing code from Medical Services Plan or hospitalization code from Discharge Abstract Database was recorded for each individual; from the date of first HCV diagnosis to last DAA treatment initiation (or the same date for matched untreated individual). Categories were created using 25th, 50th and 75th percentiles based on average number of healthcare visits coded for the overall study population
[c]Other category included residents of British Columbia with ethnic ancestry defined as White, Indigenous, Black, Latin American, Pacific Islander, Central/West Asian, Filipino, Southeast Asian or Other
[d]Other/unknown category included HCV genotypes 2, 3, other and unknown genotypes

© 2025 Jeong D et al. *JAMA Network Open*.

eTable 5. Adjusted subdistributional hazard ratios for the association between direct-acting antivirals and the risk of incident extrahepatic manifestations, overall and stratified by age

	Overall study population Adjusted sHR[a] (95% CI)	Born on or before 1959 Adjusted sHR[a] (95% CI)	Born after 1959 Adjusted sHR[a] (95% CI)	HCV genotype 1 Adjusted sHR[a] (95% CI)	HCV genotype 3 Adjusted sHR[a] (95% CI)
**CKD & ESKD**					
Untreated	Ref	Ref	Ref	Ref	Ref
Treated & SVR	0.70 (0.61-0.80)	0.74 (0.62-0.88)	0.62 (0.49-0.80)	0.73 (0.60-0.89)	0.58 (0.41-0.82)
Treated & No-SVR	0.46 (0.20-1.06)	0.64 (0.26-1.58)	-	0.38 (0.12-1.22)	1.22 (0.49-3.07)
**T2DM**					
Untreated	Ref	Ref	Ref	Ref	Ref
Treated & SVR	1.29 (1.04-1.60)	1.09 (0.81-1.47)	1.59 (1.16-2.20)	1.14 (0.84-1.54)	1.42 (0.88-2.31)
Treated & No-SVR	-	-	-	-	-
**Stroke**					
Untreated	Ref	Ref	Ref	Ref	Ref
Treated & SVR	0.86 (0.76-1.06)	0.91 (0.71-1.17)	0.73 (0.50-1.06)	0.82 (0.62-1.09)	1.09 (0.64-1.84)
Treated & No-SVR	-	-	-	-	-
**MACE**					
Untreated	Ref	Ref	Ref	Ref	Ref
Treated & SVR	0.72 (0.64-0.81)	0.77 (0.66-0.90)	0.62 (0.50-0.77)	0.73 (0.62-0.87)	0.79 (0.59-1.08)
Treated & No-SVR	0.46 (0.23-0.95)	0.74 (0.35-1.54)	-	0.45 (0.17-1.18)	0.56 (0.15-2.03)
**NCD**					
Untreated	Ref	Ref	Ref	Ref	Ref
Treated & SVR	0.67 (0.58-0.79)	0.68 (0.57-0.82)	0.61 (0.46-0.80)	0.65 (0.52-0.81)	0.80 (0.55-1.16)
Treated & No-SVR	0.74 (0.36-1.51)	0.91 (0.40-2.06)	-	0.66 (0.25-1.75)	0.75 (0.19-3.02)

Abbreviations: sHR, cause-specific hazard ratio; CI, confidence interval; CKD, chronic kidney disease; ERSD, end-stage kidney disease; MACE, major adverse cardiac events; NCD, neurocognitive disorders; SVR, sustained virologic response; T2DM, diabetes mellitus.

[a]Adjusted subdistributional hazard ratios were obtained from IPTW for ATE weighted Fine-Gray subdistributional hazards models adjusted for sex (Male, Female), birth year, age at first HCV diagnosis (years), ethnicity (East Asian, South Asian, Other), material deprivation quintiles, social deprivation quintiles, HCV genotype (genotype 1, other), baseline diagnosis of HBV infection, HIV infection, hypertension, statin use, obesity, NAFLD, prevalent T2DM (except in model for incident T2DM), cirrhosis, major mental illness, alcohol use disorder, drug dependence disorder, injection drug use, and opioid agonist therapy.

© 2025 Jeong D et al. *JAMA Network Open*.

eTable 6. Adjusted cause-specific and subdistributional hazard ratios for the association between direct-acting antivirals and the risk of incident extrahepatic manifestations, in the overall study population excluding IFN-experienced individuals

	Adjusted csHR (95% CI)	Adjusted sHR (95% CI)
**CKD & ESKD**		
Untreated	Ref	Ref
Treated & SVR	0.54 (0.46-0.63)	0.68 (0.59-0.79)
Treated & No-SVR	0.50 (0.20-1.25)	0.52 (0.21-1.29)
**T2DM**		
Untreated	Ref	Ref
Treated & SVR	1.03 (0.82-1.28)	1.27 (1.01-1.58)
Treated & No-SVR	-	-
**Stroke**		
Untreated	Ref	Ref
Treated & SVR	0.69 (0.56-0.86)	0.89 (0.72-1.10)
Treated & No-SVR	-	-
**MACE**		
Untreated	Ref	Ref
Treated & SVR	0.62 (0.55-0.70)	0.76 (0.67-0.86)
Treated & No-SVR	0.34 (0.14-0.87)	0.36 (0.14-0.91)
**NCD**		
Untreated	Ref	Ref
Treated & SVR	0.53 (0.45-0.62)	0.68 (0.58-0.80)
Treated & No-SVR	0.81 (0.38-1.73)	0.85 (0.40-1.81)

Abbreviations: sHR, cause-specific hazard ratio; CI, confidence interval; CKD, chronic kidney disease; ERSD, end-stage kidney disease; MACE, major adverse cardiac events; NCD, neurocognitive disorders; SVR, sustained virologic response; T2DM, diabetes mellitus.

[a]Adjusted cause-specific and subdistributional hazard ratios were obtained from IPTW for ATE weighted multivariable hazards models adjusted for sex (Male, Female), birth year, age at first HCV diagnosis (years), ethnicity (East Asian, South Asian, Other), material deprivation quintiles, social deprivation quintiles, HCV genotype (genotype 1, other), baseline diagnosis of HBV infection, HIV infection, hypertension, statin use, obesity, NAFLD, prevalent T2DM (except in model for incident T2DM), cirrhosis, major mental illness, alcohol use disorder, drug dependence disorder, injection drug use, and opioid agonist therapy.

© 2025 Jeong D et al. *JAMA Network Open*.

eTable 7. Adjusted cause-specific and subdistributional hazard ratios for the association between direct-acting antivirals and the risk of incident extrahepatic manifestations, in the overall study population comparing treated vs untreated groups

	Adjusted csHR (95% CI)	Adjusted sHR (95% CI)
**CKD & ESKD**		
Untreated	Ref	Ref
Treated	0.56 (0.48-0.64)	0.70 (0.61-0.81)
**T2DM**		
Untreated	Ref	Ref
Treated	1.02 (0.82-1.27)	1.27 (1.03-1.57)
**Stroke**		
Untreated	Ref	Ref
Treated	0.64 (0.52-0.78)	0.83 (0.67-1.01)
**MACE**		
Untreated	Ref	Ref
Treated	0.58 (0.51-0.65)	0.71 (0.63-0.80)
**NCD**		
Untreated	Ref	Ref
Treated	0.54 (0.46-0.63)	0.69 (0.59-0.80)

Abbreviations: sHR, cause-specific hazard ratio; CI, confidence interval; CKD, chronic kidney disease; ERSD, end-stage kidney disease; MACE, major adverse cardiac events; NCD, neurocognitive disorders; SVR, sustained virologic response; T2DM, diabetes mellitus.

[a]Adjusted cause-specific and subdistributional hazard ratios were obtained from IPTW for ATE weighted multivariable hazards models adjusted for sex (Male, Female), birth year, age at first HCV diagnosis (years), ethnicity (East Asian, South Asian, Other), material deprivation quintiles, social deprivation quintiles, HCV genotype (genotype 1, other), baseline diagnosis of HBV infection, HIV infection, hypertension, statin use, obesity, NAFLD, prevalent T2DM (except in model for incident T2DM), cirrhosis, major mental illness, alcohol use disorder, drug dependence disorder, injection drug use, and opioid agonist therapy

© 2025 Jeong D et al. *JAMA Network Open*.

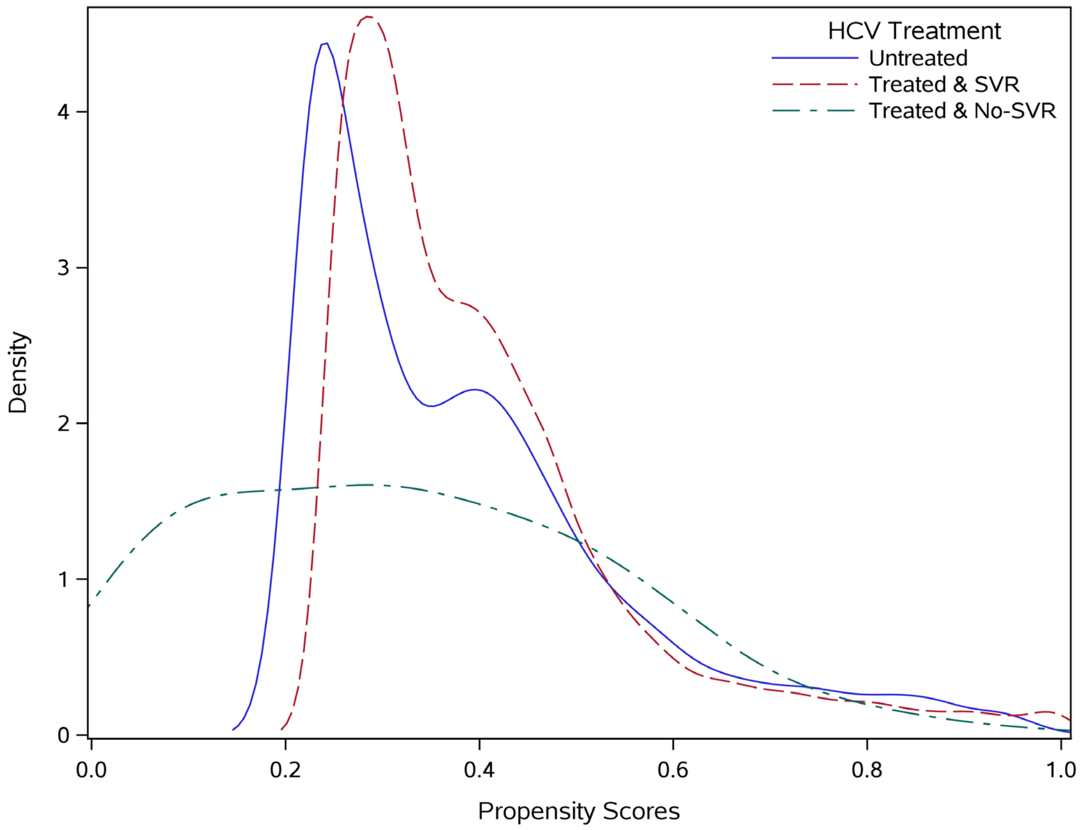

eFigure 1. Propensity score density plot of overall study population

© 2025 Jeong D et al. *JAMA Network Open*.
