# Supplementary material for: Direct-Acting Antivirals and Risk of Hepatitis C Extrahepatic Manifestations
Source: JAMA Netw Open. 2025 Jun 11;8(6):e2514631. doi: 10.1001/jamanetworkopen.2025.14631 (PMC12159775; doi:10.1001/jamanetworkopen.2025.14631)
Supplement: Supplement 2. — Data Sharing Statement [file jamanetwopen-e2514631-s002.pdf]

Data Sharing Statement

Jeong. Direct-Acting Antivirals and Risk of Hepatitis C Extrahepatic Manifestations. *JAMA Netw Open*. Published June 11, 2025. doi:10.1001/jamanetworkopen.2025.14631

Data
Data available: No

Additional Information
Explanation for why data not available: The study is based on data contained in various provincial registries and databases. Access to data could be requested through the BC Centre for Disease Control Institutional Data Access for researchers who meet the criteria for access to confidential data. Requests for the data may be sent to datarequest@bccdc.ca.
